# Locus of control, self-control, and health outcomes^☆^

**DOI:** 10.1016/j.ssmph.2023.101566

**Published:** 2023-11-24

**Authors:** Ferdi Botha, Sarah C. Dahmann

**Affiliations:** a The University of Melbourne, Melbourne Institute: Applied Economic & Social Research; b ARC Centre of Excellence for Children and Families over the Life Course; c Institute of Labor Economics (IZA

**Keywords:** locus of control, Self-control, Health, Health behavior

## Abstract

We provide the first empirical evidence on the direct link between locus of control and self-control, and how they interact in explaining a range of health outcomes. Using rich Australian survey data, we find that, while the two traits are distinct constructs, a greater internal locus of control is associated with higher self-control. The association between locus of control and health is reduced once we control for self-control, suggesting that self-control mediates at least part of this relationship. Finally, an internal locus of control amplifies the beneficial effects of self-control particularly for physical health.

## Highlights

• We study the link between locus of control, self-control, and health outcomes.
• A greater internal locus of control is associated with higher self-control.
• Self-control mediates part of the relationship between locus of control and health.
• An internal locus of control strengthens the beneficial effects of self-control.

## 1 Introduction

Globally, noncommunicable diseases are the leading cause of death and there has been a rise in most risk factors for their development (WHO, 2018). For example, the worldwide prevalence of obesity has nearly tripled between 1975 and 2016 (Bentham et al., 2017). Many risk factors for noncommunicable diseases are preventable through, for example, increased physical activity, better nutrition, and limited consumption of alcohol and tobacco. However, it is unclear why some people choose to adopt such healthier behaviors while others do not. Given the importance of good health for individuals, their families, but also society—with a healthy society contributing to population productivity and lowering the vast economic costs associated with the burden of disease—it is critical to understand the behavioral underpinnings of people's health.

This paper investigates the joint role for people's health of locus of control and self-control—two personality traits that independently have been shown to be key for understanding people's health and health behavior. Locus of control describes the belief about the extent to which life events are due to own actions—characterizing people with an internal locus of control—rather than outside forces beyond one's control—characterizing people with an external locus of control (Rotter, 1966). There is ample evidence of a link between a greater internal locus of control and improved physical health, health behaviors, and psychological wellbeing (Awaworyi Churchill et al., 2020; Buddelmeyer & Powdthavee, 2016; Cobb-Clark et al., 2014; Hoffmann & Risse, 2020; Kesavayuth et al., 2020).

Self-control, in turn, is often described as the ability to override automatic impulses (Boals et al., 2011). It helps people resist temptation and achieve their longer-term goals. Consequently—and similar to internal locus of control—greater self-control has been linked to a wide range of health outcomes. Greater self-control is associated, for example, with a healthier lifestyle and physical and mental wellbeing (Boals et al., 2011; Cobb-Clark et al., 2022, 2023a; Moffitt et al., 2011; Tangney et al., 2004).

Thus, while locus of control and self-control are distinct concepts (Ajzen, 2002; Peterson & Stunkard, 1992), their importance in predicting people's health is common to both. In addition, they are also conceptually related (see Bandura, 1977; Rosenbaum, 1980). Yet, large-scale *empirical* evidence on the link between locus of control and self-control is surprisingly limited within the broader psychology and economics literature. Twenge et al.’s (2004) meta-analysis identifies some small studies from the 1960's and 1970's suggesting that an external locus of control is associated with decreased self-control. Yet, to our knowledge only a limited number of studies have estimated the relationship between the two constructs, reporting correlations between internal locus of control and self-control ranging from 0.24 to 0.40 (Flores et al., 2020; Gough, 1974; Richards, 1985; Rohrbeck et al., 1991; Rosenbaum, 1980). Flores et al. (2020) also find that greater internal locus of control and self-control are independently associated with better mental health outcomes. All these studies are, however, based on small and non-representative samples and therefore limited in scope.

We make several important contributions to this literature. First, to our knowledge, this study is the first to empirically test the theorized association between locus of control and self-control in a population representative survey. The Household, Income and Labour Dynamics in Australia (HILDA) survey—as the first population representative sample to include both concepts—provides measures of both locus of control and self-control in its 2019 wave. Our empirical results indeed support the theory that a more internal locus of control is related to greater self-control. At the same time, however, we demonstrate empirically that locus of control and self-control are distinct concepts. This insight invalidates the approach taken in some studies of interpreting measures of locus of control to capture self-control or vice versa. For example, when studying the relationship between self-control and obesity, Fan and Jin (2014) measure self-control using the Rotter (1966) locus of control scale and Stutzer and Meier (2016) measure self-control using the Pearlin and Schooler's (1978) Mastery Scale, which captures locus of control.

Second, we investigate whether self-control is a channel through which locus of control affects people's physical and psychological wellbeing. Cobb-Clark (2015, p. 5) argues that “if self-control is enhanced by [an internal locus of control] and is diminished by [an external locus of control], then self-control may be another pathway linking locus of control to many of life's outcomes”. Focusing specifically on physical health, health behaviors, and mental health—which both locus of control as well as self-control have independently been linked to—we provide the first empirical investigation into this hypothesis. Our findings demonstrate that at least part of the relationship of most health outcomes with locus of control is explained by the association between locus of control and self-control. Self-control thus expands the set of mechanisms through which locus of control may affect health—corroborating the hypothesis outlined in Cobb-Clark (2015).

Finally, we investigate how locus of control and self-control—both having been shown to independently impact health—interact in predicting people's health. We show that, while having high self-control is related to better health, having an internal locus of control amplifies the beneficial health impacts of self-control, particularly for physical health. These findings suggest that there could be crucial benefits for policymakers of targeting both locus of control and self-control simultaneously when addressing population health: Policies to enhance self-control for the benefit of improving individual health outcomes may likely be of limited success among those with an external locus of control. Conversely, intervention programs designed to improve both people's self-control and their locus of control could yield great efficiency gains.

The remainder of the paper is organized as follows: Section 2 outlines theoretical considerations around the relationship between the two personality traits and how they may interact in predicting health outcomes. Section 3 provides the details of our data, and we describe the empirical link between locus of control and self-control in Section 4. Next, we focus on both traits' relationship with health outcomes by first investigating whether self-control mediates the relationship between locus of control and health outcomes (Section 5) and second estimating the joint effect of a more internal locus of control and greater self-control for people's health (Section 6). Finally, Section 7 concludes and discusses the implications of our findings.

## 2 Theoretical considerations

Locus of control is the belief about whether life events are due to own actions (internal) or due to outside forces beyond your control (external) (Rotter, 1966), while self-control refers to the “ability to override automatic impulses” (Boals et al., 2011, p. 1050), often to achieve longer-term goals. Thus, they are two different constructs. For example, Almlund et al. (2011) conceptualize both constructs as belonging to different personality factors: self-control relates to conscientiousness, while locus of control relates to neuroticism. Similarly, Bandura (1977) criticizes the fact that locus of control is often treated as analogous to self-efficacy—the degree of confidence in the ability to exert self-control. He argues that “[p]erceived self-efficacy and beliefs about the locus of causality must be distinguished” (p. 204).

While being distinct constructs, locus of control and self-control are, however, conceptually related. Bandura (1977), for example, argues that a person's self-control must depend on the belief that they have some degree of control over what happens to them. Conceptually, an individual's self-control should thus depend on their locus of control. As Cobb-Clark (2015) argues, this relationship lends itself to hypothesizing that self-control may be a mediator in linking locus of control to many of life's outcomes, including health.

At the same time, the effect of self-control on people's health behaviors—and thereby health outcomes—could also depend on their locus of control. Rosenbaum (1980, p. 111) argues that “before a person applies any specific self-controlling skill he must believe that he can control his own behavior without outside help”. Thus, individuals may consider it more worthwhile to exercise self-control if they believe that their actions can make a difference.

In the next sections, we conduct an empirical investigation into each of these hypothesized relationships individually. Our analyses are descriptive rather than causal; we therefore rely on the theoretical considerations outlined in this section to guide our interpretation of the direction of effects. For example, we do not consider the reverse relationship in which locus of control could mediate the effect of self-control on people's health. Similarly, as it aligns with theory, we interpret any joint effects of locus of control and self-control in predicting people's health as support for locus of control acting as a moderator, even though empirically either could of course be the moderator in the interaction.

## 3 Data

We base our empirical analyses on data from the Household, Income and Labour Dynamics in Australia (HILDA) survey. HILDA is a nationally representative household panel, started in 2001 and surveying more than 17,000 Australians annually. The survey provides rich information on a broad range of people's life aspects, including, for example, their socioeconomic conditions, labor market history, relationships, and health and wellbeing. Importantly, in its 2019 wave HILDA includes measures of both locus of control and self-control as part of the Self-Completion Questionnaire (SCQ), making it the ideal dataset for the purpose of this study.

**Measuring locus of control and self-control.** In HILDA, locus of control is elicited through seven items from the Pearlin and Schooler's (1978) Mastery Scale, which is commonly used among economists to capture locus of control beliefs (e.g., Buddelmeyer & Powdthavee, 2016; Cobb-Clark et al., 2014; Cobb-Clark & Schurer, 2013). Individuals indicate how much they agree with the statements in the items on scale from 1 (“strongly disagree”) to 7 (“strongly agree”). The items include, for example, having little control over things that happen to them, feeling helpless in dealing with problems, feeling pushed around in life, or believing that what happens to them in the future mostly depends on them. For the full list of items, see Table A.1. We reverse responses to some of the items, such that higher scores represent greater perceived control, i.e., a more internal locus of control. To calculate an overall score, we take the average across all items. Thus, the final score ranges from 1 to 7, with higher values indicating a more internal locus of control. Cronbach's alpha for this locus of control scale is 0.84, suggesting excellent reliability (see Table A.3). For ease of interpretation, we standardize the final score that we use across all empirical analyses to have mean zero and standard deviation one, such that effect sizes can be interpreted in terms of standard deviations.

HILDA includes the Brief Self-Control Scale (BSCS; see Tangney et al., 2004)—a 13-item battery of questions measuring general trait self-control, that is highly correlated with the more extensive full 36-item scale. People indicate how well each of the 13 items describes how they usually are on a scale from 1 (“not at all”) to 5 (“very well”). The items include, for example, whether they are good at resisting temptation, often act without thinking, can work effectively towards long-term goals, or have a hard time breaking bad habits. For the full list of items, see Table A.2. Again, we reverse responses to some items, such that higher scores represent greater self-control, and take the average across all 13 items as a measure of people's self-control, which ranges from 1 to 5. Previous research has demonstrated high internal consistency and test-retest reliability for this measure of trait self-control (Bertrams & Dickhäuser, 2009; Tangney et al., 2004), which we confirm based on our data with a Cronbach's alpha of 0.84 (see Table A.4). Again, for all empirical analyses, we standardize the final score to have mean zero and standard deviation one, such that effect sizes of both locus of control and self-control can easily be compared within analyses.

Both locus of control and self-control measures are taken from HILDA's 2019 wave. Thus, locus of control, self-control, and all health outcomes (see next paragraph) are measured contemporaneously; our empirical analysis is thus descriptive in nature. As such, our empirical evidence provides an understanding of population-wide patterns in the interrelationship between locus of control, self-control, and people's health. While not causal, such descriptive evidence can be valuable guidance for generating hypotheses, understanding mechanisms, and interpreting findings, and provide a target for practitioners and researchers to focus their efforts on (Loeb et al., 2017). In addition, as personality traits, both locus of control and self-control are malleable in childhood and throughout adolescence but are generally assumed to be stable throughout adulthood. Indeed, this is confirmed by Cobb-Clark and Schurer (2013) for locus of control and by Cobb-Clark, Kong, and Schildberg-Hörisch (2023) for trait self-control who find that both traits are relatively stable over several years for adults and are not related to key life events.

This stability of both traits goes a long way in addressing potential concerns of endogeneity, as it reduces the risk of a spurious correlation (e.g., locus of control and self-control both being impacted by the same life event) or reverse causality in our analyses focusing on health outcomes (e.g., health shocks impacting the traits). Nonetheless, other sources of endogeneity may still be present, making our empirical analysis descriptive in nature. Also, as both are self-reported, measurement error may plague the locus of control and self-control scores. For locus of control we have repeated measurements, such that, in additional robustness checks, we can address potential measurement error in two ways: First, we test the sensitivity of our results to constructing locus of control as the average across all waves with the trait available for each individual rather than just using the 2019 observation. The HILDA survey has included the Pearlin and Schooler (1978) Mastery Scale in 2003, 2004, 2007, 2011, 2015, and 2019. In addition to reducing measurement error, this approach also mitigates any concerns of short-term changes in locus of control (see, e.g., Preuss & Hennecke, 2018, who find that unemployment shocks lower locus of control in the short-run but have no lasting impact in the long-run). Second, we use the previous locus of control measure from 2015 to instrument locus of control in 2019. For self-control, we unfortunately do not have multiple observations as the BSCS was included in HILDA's 2019 wave for the first time. With the item battery including 13 questions, however, we expect measurement error to be less of a concern than for locus of control which is based on a shorter, 7-item scale. Finally, measurement error—and therefore attenuation bias in the coefficients—can also stem from using a simple average of all items to construct the locus of control and the self-control measures (see Piatek & Pinger, 2016). We therefore also use loadings obtained from factor analyses instead to confirm that the results remain consistent.

**Health outcomes.** HILDA surveys people's physical and mental wellbeing in detail, which allows us to study a wide range of aspects of people's health including (i) their overall and physical health; (ii) health behaviors; and (iii) their mental health and wellbeing. We capture overall and physical health through people's self-rating of their health in general on a scale from 1 (poor) to 5 (excellent), as well as two measures derived from the SF-36 Health Questionnaire: the Physical Component Summary (PCS), (standardized to mean zero and standard deviation one) and the general health subscale (ranging from 0, low, to 100, high). In addition, we use information on people's Body Mass Index (BMI) to construct indicators for being overweight (BMI ≥25) and for being obese (BMI ≥30).

Health behaviors include whether people are inactive, i.e., do not at all participate in physical activity for at least 30 min, whether they currently smoke and, if so, the number of cigarettes per week, whether they drink alcohol at least once a week, and whether they have three or more alcoholic drinks per occasion. Some of these behaviors can be directly related to the other health outcomes, e.g., inactivity may lead to being overweight. Therefore, in addition to being outcomes worth studying, they likely further serve as potential mechanisms for (some of) the other health outcomes.

Finally, we measure people's mental health and wellbeing in various ways. We rely on the SF-36 for measures of the Mental Component Summary (MCS) score (standardized to mean zero and standard deviation one) and the Mental Health subscale (ranging from 0, low, to 100, high). In addition, we calculate an indicator for psychological distress that equals one if respondents' Kessler-10 score is greater or equal to 30, indicating high or very high distress (see Kessler et al., 2002). To measure wellbeing, we rely on people's reported satisfaction with both life in general and with health, each reported on a scale from 0 (“very dissatisfied”) to 10 (“very satisfied”).

A full list of all variables and their definitions is provided in Table A.5 and their summary statistics in Table A.6.

**Sample.** We base our analyses on data from the 2019 wave of HILDA, which includes 17,462 respondents, of whom 16,150 completed the SCQ. Of these potential observations, we exclude 852 respondents with missing information on either locus of control or self-control. We exclude a further 10 observations with incomplete information on the set of control variables that we include in our analyses: age, gender, education, immigrant status, Indigenous status, and state of residence. Our final analysis sample thus consists of 15,288 respondents. In Table A.7 we compare means across personality traits, health outcomes, and basic demographics in our final analysis sample and the full sample (before sample restrictions are applied). Naturally, some of these variables are only available for SCQ respondents and those who provided the relevant information. Demographic variables, however, are available for almost everybody. Only three out of a total of 40 variables reveal a statistically significant difference, which can be expected given this large number of hypotheses tests: Our final sample includes slightly fewer respondents with educational attainment of year 11 or less and conversely slightly more respondents with a Bachelor degree or higher. It also includes slightly fewer Indigenous respondents. However, the differences are economically small. Overall, our analysis sample is thus representative of the Australian population across a range of demographics and characteristics. Nonetheless, in sensitivity analyses we also estimate weighted regressions that account for SCQ non-response.

## 4 The link between locus of control and self-control

We start our empirical analysis by investigating the link between locus of control and self-control. An exploratory factor analysis of the total of 20 items from both the locus of control scale and the BSCS reveals two factors with an eigenvalue above one. Restricting the factor analysis to two factors in a next step, we report the resulting factor loadings with oblique rotation in Table 1. The loadings clearly support the distinction of the two concepts: All locus of control items load on the same factor (Factor 1), with the highest loadings among items 2–4 (between 0.77 and 0.82) and the lowest loading for item 6 at 0.39. Similarly, all self-control items load on the same factor (Factor 2), with the highest factor loading of 0.67 for item 5 and the lowest loading for item 11, at 0.30. Figure A.1 visualizes these factor loadings, again emphasizing the distinctiveness of the two factors. Thus, our findings demonstrate that not only theoretically, but also empirically, locus of control and self-control are distinct concepts.

**Table 1:** Rotated factor loadings.

Item	Question	Factor 1	Factor 2	Uniqueness
LOC1	I have little control over the things that happen to me.^a^	**0.6864**	−0.0430	0.5498
LOC2	There is really no way I can solve some of problems I have.^a^	**0.8000**	−0.0431	0.3849
LOC3	There is little I can do to change many of the important things in my life.^a^	**0.8193**	−0.0859	0.3759
LOC4	I often feel helpless in dealing with the problems of life.^a^	**0.7672**	0.0834	0.3550
LOC5	Sometimes I feel that I'm being pushed around in life.^a^	**0.6791**	0.0898	0.4836
LOC6	What happens to me in the future mostly depends on me.	**0.3913**	−0.0572	0.8609
LOC7	I can do just about anything I really set my mind to do.	**0.4948**	0.0224	0.7461
BSCS1	I am good at resisting temptation.	−0.0650	**0.5899**	0.6775
BSCS2	I have a hard time breaking bad habits.^a^	0.0131	**0.5314**	0.7121
BSCS3	I am lazy.^a^	0.0753	**0.4987**	0.7166
BSCS4	I say inappropriate things.^a^	0.0296	**0.5040**	0.7335
BSCS5	I do certain things that are bad for me, if they are fun.^a^	−0.1132	**0.6710**	0.5958
BSCS6	I refuse things that are bad for me.	−0.0787	**0.4696**	0.8019
BSCS7	I wish I had more self-discipline.^a^	−0.0163	**0.6162**	0.6278
BSCS8	People would say I have iron self-discipline.	−0.0658	**0.4484**	0.8175
BSCS9	Pleasure and fun sometimes keep me from getting work done.^a^	−0.0543	**0.5266**	0.7419
BSCS10	I have trouble concentrating.^a^	0.2224	**0.4741**	0.6441
BSCS11	I can work effectively towards long-term goals.	0.2507	**0.3018**	0.7875
BSCS12	Sometimes I cannot stop myself from doing something even if I know it is wrong.^a^	0.0646	**0.6001**	0.6057
BSCS13	I often act without thinking through all the alternatives.^a^	0.0970	**0.5311**	0.6686

*Notes:* HILDA wave 19, analysis sample with 15,288 observations. Loadings from factor analysis restricted to two factors after oblique rotation. LOC indicates items from the Pearlin and Schooler's (1978) Mastery Scale to measure locus of control; BSCS indicates items from the Brief Self-Control Scale (Tangney et al., 2004) to measure self-control. Responses to items marked with.

a are reversed. Factor loadings with an absolute value above 0.3 in bold.

Given that self-control and locus of control are distinct concepts, we next consider the question of whether, and to what extent, locus of control predicts self-control to investigate their relationship. We estimate an OLS regression equation of the form:

(1) $${SC}_{i}=\alpha +\beta {LOC}_{i}+\gamma X_{i}+\varepsilon_{i}\text{,}$$

where for person *i, SC*_*i*_ is their self-control score, *LOC*_*i*_ is their locus of control score, ***X***_*i*_ is a vector of control variables, and $\varepsilon_{i}$ is an error term. In a first step, we estimate the model including only locus of control as regressor without any further control variables, such that β captures the unconditional correlation between locus of control and self-control (as both measures are standardized). In a second step, we obtain the correlation conditional on key demographic characteristics by re-estimating the model including the full set of controls: gender, age, education, immigrant status, Indigenous status, and state of residence fixed effects (see Tables A.5 and A.6 for definitions and summary statistics, respectively). We present the results of both estimations in Table 2.

**Table 2:** Self-control regressed on locus of control.

	(1)	(2)
LOC	0.357***	0.366***
	(0.008)	(0.008)
Male		−0.151***
		(0.014)
*Age (reference category: 15–24)*		
25-34		0.109***
		(0.027)
35-44		0.231***
		(0.029)
45-54		0.359***
		(0.028)
55-64		0.516***
		(0.027)
65+		0.751***
		(0.026)
*Education (reference category: Year 11 and below)*		
Year 12		−0.010
		(0.025)
Cert III or IV, or (Adv.) Diploma		0.026
		(0.020)
Bachelor degree or higher		0.089***
		(0.022)
*Immigrant status (reference category: Australian-born)*		
Main English speaking		−0.008
		(0.025)
Other migrant		0.276***
		(0.023)
Indigenous		0.024
		(0.044)
Adj. R^2^	0.127	0.215
Obs.	15,288	15,288

*Notes:* HILDA wave 19, analysis sample. OLS regressions with self-control as outcome variable. In addition, specifications in all columns control for a constant and specification in column (2) controls for a maximum set of fixed effects for the state of residence. Robust standard errors in parentheses. *p < 0.1, **p < 0.05, ***p < 0.01.

We focus on this parsimonious set of control variables as they are arguably exogenous, except for education and state of residence. While state of residence is a choice, it is unlikely to be systematically linked to locus of control or self-control. The completion of education, in contrast, is likely affected by both. We include it nonetheless, as we are interested in capturing the contemporaneous link between the two that operates beyond education, which most people in our sample have completed (long) in the past. We refrain from including other personality traits in any of our analyses, such as the Big Five, as they are conceptually related and would thus blur the interpretation of our result (see Almlund et al., 2011, for an overview of different personality taxonomies and empirical evidence).

The locus of control parameter in column (1) denotes the unconditional correlation coefficient with self-control. The value suggests a positive correlation between locus of control and self-control of 0.357. This correlation is very close to the range between 0.37 and 0.40 found in other studies (Flores et al., 2020; Richards, 1985; Rosenbaum, 1980). Adding a set of control variables in column (2) has little effect on the locus of control coefficient with the estimate increasing only slightly to 0.366. Thus, the relationship between locus of control and self-control appears insensitive to key demographic characteristics—despite most of them being significant predictors of self-control as well.

Overall, our results suggest that a one standard deviation increase in (more internal) locus of control is associated with an increase in self-control by about 0.36–0.37 standard deviations, on average. Thus, in line with our expectations, a more internal locus of control is correlated with greater self-control. This finding implies that self-control could potentially be a mechanism through which locus of control improves people's life outcomes (Cobb-Clark, 2015), which we investigate in the next section focusing on health. At the same time, however, the moderate correlation lends further support to our finding that, even though related, the two concepts are distinct. These results are robust to using predicted factor scores for locus of control and self-control as well as weighted regressions that adjust for SCQ non-response (Table A.8).

## 5 Does the link between locus of control and health operate through self-control?

Next, we investigate whether self-control—given it is distinct from but related to locus of control—is potentially a mechanism through which locus of control improves people's health outcomes. We focus on physical health, health behaviors, and mental health, which have independently been linked to both locus of control and self-control. Specifically, we examine what proportion of the association between locus of control and the respective health outcomes can be explained by self-control.

For this purpose, we first estimate models of the form:

(2) $$H_{i}=\tilde{\alpha }+\omega {LOC}_{i}+\tilde{\gamma }X_{i}+\epsilon_{i}\text{,}$$

where *H*_*i*_ is the health outcome of interest and all other variables are defined as before. We then add self-control to the model and estimate:

(3) $$H_{i}=\bar{\alpha }+\delta {LOC}_{i}+\varphi {SC}_{i}+\bar{\gamma }X_{i}+e_{i}\text{.}$$

Thus, for each health outcome *H*_*i*_ we first regress the outcome on locus of control and a set of controls, after which we also add self-control in a subsequent model. Our focus here is on the extent to which the locus of control parameter in equation (2) changes once adjusting for self-control in equation (3).

To formalize the mediating relationships further, we estimate a mediation model similar in approach to that of Tubeuf et al. (2012), which allows us to quantify the proportion of the relationship of locus of control with health that operates outside of self-control (the direct effect) and the proportion of locus of control that operates through self-control (the indirect effect). In our specifications, the direct effect of locus of control on health is simply δ from equation (3). The indirect effect is obtained by multiplying β from equation (1) with φ from equation (3). Thus, the proportion of the relationship between locus of control and health that operates through self-control is βφ. The total, i.e., direct plus indirect, effect of locus of control on health is therefore δ+βφ. As the sample varies slightly across outcomes, we use outcome-specific estimates of β that are obtained by re-estimating equation (1) using only the available observations for each health outcome considered; they are very close to β reported in Table 2, however.

The main results are reported in Table 3. They are in line with existing literature (e.g., Cobb-Clark et al., 2014; Etilé et al., 2021; Hoffmann & Risse, 2020; Verme, 2009), confirming that an internal locus of control is generally associated with better physical and mental health, greater wellbeing, and increased probabilities of being physically active and being a non-smoker. Effect sizes are moderate to substantial with the relative effect sizes ranging between 10 and 20 percent for most outcomes, up to 42 percent (physical inactivity) and 96 percent (psychological distress). One exception relates to alcohol consumption, where an internal locus of control is associated with an increased (rather than decreased) likelihood of alcohol consumption and excessive drinking. While contrary to the otherwise beneficial health impacts of locus of control, these findings are consistent with previous evidence: Caliendo and Hennecke (2022) and Cobb-Clark et al. (2014) also find that a more internal locus of control is associated with greater alcohol consumption—attributable at least in part to differences in the social activities.

**Table 3:** Health outcomes regressed on locus of control and self-control.

	(1)	(2)	(3)	(4)	(5)	(6)	(7)	(8)	(9)	(10)
Panel A: Overall and Physical Health										
	Self-rated health		PCS		General health		Overweight		Obese	
LOC	0.349***	0.291***	0.261***	0.244***	9.926***	8.332***	−0.019***	0.004	−0.036***	−0.016***
	(0.007)	(0.008)	(0.008)	(0.009)	(0.161)	(0.175)	(0.004)	(0.004)	(0.004)	(0.004)
	[0.104]	[0.087]	.	.	[0.150]	[0.126]	[-0.031]	[0.007]	[-0.133]	[-0.061]
SC		0.160***		0.045***		4.382***		−0.065***		−0.054***
		(0.008)		(0.008)		(0.172)		(0.004)		(0.004)
		[0.048]		.		[0.066]		[-0.106]		[-0.200]
$\chi^{2}$ test	.	340.6***	.	31.1***	.	498.7***	.	194.9***	.	166.4***
Adj. R^2^	0.233	0.254	0.272	0.274	0.264	0.298	0.067	0.081	0.044	0.056
Obs.	15,189	15,189	15,000	15,000	15,151	15,151	14,812	14,812	14,812	14,812
**Panel B: Health Behaviors**										
	Inactive		Smoking		Number of cigarettes		Alcohol: weekly		Alcohol: 3+ drinks	
LOC	−0.050***	−0.049***	−0.028***	−0.008**	−2.407**	−1.608	0.048***	0.074***	0.008*	0.041***
	(0.003)	(0.003)	(0.003)	(0.003)	(1.171)	(1.230)	(0.004)	(0.004)	(0.004)	(0.005)
	[-0.420]	[-0.406]	[-0.176]	[-0.049]	[-0.034]	[-0.023]	[0.112]	[0.171]	[0.017]	[0.084]
SC		−0.005		−0.056***		−2.600**		−0.070***		−0.089***
		(0.003)		(0.003)		(1.272)		(0.004)		(0.005)
		[-0.038]		[-0.349]		[-0.037]		[-0.163]		[-0.185]
$\chi^{2}$ test	.	2.4	.	254.8***	.	4.2**	.	238.7***	.	292.6***
Adj. R^2^	0.061	0.061	0.076	0.094	0.090	0.091	0.080	0.096	0.093	0.118
Obs.	15,234	15,234	15,178	15,178	2319	2319	15,164	15,164	12,154	12,154
**Panel C: Mental Health and Wellbeing**										
	MCS		Mental health		Psychological distress		Life satisfaction		Health satisfaction	
LOC	0.588***	0.525***	10.581***	9.490***	−0.192***	−0.174***	0.635***	0.577***	0.768***	0.664***
	(0.007)	(0.008)	(0.132)	(0.144)	(0.003)	(0.003)	(0.013)	(0.014)	(0.016)	(0.018)
	.	.	[0.146]	[0.131]	[-0.964]	[-0.872]	[0.080]	[0.072]	[0.107]	[0.093]
SC		0.176***		2.997***		−0.050***		0.158***		0.285***
		(0.008)		(0.139)		(0.003)		(0.012)		(0.017)
		.		[0.041]		[-0.253]		[0.020]		[0.040]
$\chi^{2}$ test	.	405.0***	.	381.5***	.	224.2***	.	159.9***	.	256.8***
Adj. R^2^	0.378	0.403	0.371	0.393	0.267	0.280	0.216	0.226	0.197	0.214
Obs.	15,000	15,000	15,233	15,233	15,256	15,256	15,283	15,283	15,281	15,281

*Notes:* HILDA wave 19, analysis sample. OLS regressions. All regressions control for gender, age (in categories), education (in categories), migrant status, Indigenous status, state of residence fixed effects and a constant. Robust standard errors in parentheses. Relative effect sizes, where relevant, are in brackets. $\chi^{2}$ test is a test of the null hypothesis that the locus of control coefficients from the initial (without self-control) and subsequent (with self-control) model are not statistically different from each other. *p < 0.1, **p < 0.05, ***p < 0.01.

Once we add self-control to the model, we find that—as expected—greater self-control is significantly related to improved physical health, better mental health and wellbeing, and lower likelihood of unhealthy behaviors (cf., e.g., Stutzer & Meier, 2016; Strulik, 2018; Johnston et al., 2021, Cobb-Clark et al., 2022). Importantly, the inclusion of self-control also impacts the coefficient estimate of locus of control: In almost all cases, adding self-control to the model reduces the locus of control coefficient in absolute terms, suggesting that self-control mediates part of the relationship between locus of control and health. A likelihood ratio test indicates that these changes are statistically significant across almost all outcomes (with physical inactivity being the only exception).

To formally investigate the extent of this mediation, we use these estimations to decompose the total effect of locus of control on health outcomes into its direct and indirect (via self-control) effect in Table 4, following Tubeuf et al.’s (2012) mediation model approach. Self-control mediates a particularly large part of the influence of locus of control on smoking behavior and the likelihood of being obese or overweight: The indirect effect accounts for more than half (54.5%, obese), almost three-quarters (72.2%, smoking), or even all (overweight) of the total effect. Self-control also mediates part of the association with locus of control for the remaining physical health outcomes (ranging from 6.3% to 16.7%) as well as all mental health and wellbeing outcomes (9.0%–13.5%), albeit to a much smaller degree compared to body weight and smoking behavior.

**Table 4:** Direct effects of locus of control and indirect effects of locus of control via self-control on health outcomes.

	(1)	(2)
	Direct/Total	Indirect/Total
**Overall and physical health**		
Self-rated health	83.269***	16.731***
PCS	93.687***	6.313***
General health	83.945***	16.055***
Overweight	−22.585	122.585***
Obese	45.547***	54.453***
**Health behaviors**		
Inactive	96.680***	3.320
Smoking	27.786***	72.214***
Number of cigarettes	66.806	33.194
Alcohol: weekly	152.568***	−52.568***
Alcohol:3+ drinks	496.051	−396.051
**Mental health and wellbeing**		
MCS	89.157***	10.843***
Mental health	89.690***	10.310***
Psychological distress	90.422***	9.548***
Life satisfaction	90.928***	9.072***
Health satisfaction	86.497***	13.503***

*Notes:* HILDA wave 19, analysis sample. Columns (1) and (2) report the direct and indirect effects, respectively, as a proportion of the total effect (in percent). Direct, indirect, and total effects are obtained as described in Section 5. Statistical inference for the indirect effects is based on standard errors obtained via bootstrapping with 500 replications. *p < 0.1, **p < 0.05, ***p < 0.01.

There are only two exceptions—physical inactivity and alcohol consumption. There is no evidence that self-control mediates locus of control's relationship with physical inactivity. In contrast to all other outcomes, for physical inactivity there is no significant association with self-control, and the $\chi^{2}$ test suggests no significant difference in the locus of control coefficients before and after adjusting for self-control. For alcohol consumption, we find that greater self-control is statistically significantly associated with both a reduced frequency and quantity per occasion. However, given that an internal locus of control is associated with increased, rather than reduced, alcohol consumption, self-control is not mediating this relationship. Instead, our findings of lower coefficients in columns (7) and (9) compared to (8) and (10) suggest that self-control helps to counteract the detrimental locus of control effect in the case of alcohol consumption.

Overall, consistent with Cobb-Clark's (2015) assertion, self-control indeed appears to be a mechanism that links locus of control—at least in part—to a range of outcomes. This holds particularly true for outcomes related to physical and mental health and wellbeing, however not necessarily for health behaviors except for smoking.

These findings also hold true when we additionally control for the Big Five personality traits (openness to new experiences, conscientiousness, extraversion, agreeableness, and emotional stability) that are elicited using a 36-item inventory in 2017 (see Table A.9). Despite their conceptual relationship with self-control and locus of control, coefficients are often remarkably similar, emphasizing the predictive power of our measures beyond those more commonly used personality traits (see also Cobb-Clark et al., 2022). In addition, our results are robust to dealing with measurement error: Using either the average locus of control measure across all available waves (Table A.10), using the 2015 locus of control as instrument for the 2019 measure (Table A.11), or using factor scores for locus of control and self-control (Table A.12) our results are very similar to the main findings. Our results are also robust to adjusting for non-response using SCQ weights (Table A.13).

## 6 Does an internal locus of control amplify the beneficial health effects of self-control?

The results in the previous section show that, for most outcomes, having greater self-control is associated with better health, on average. A more internal locus of control tends to be associated with better health outcomes as well. In this section, we test whether having an internal locus of control amplifies the beneficial health effects of having more self-control, in line with our theoretical considerations.

For this purpose, we estimate models of the form:

(4) $$H_{i}=\overset{˘}{\alpha }+\mu I{LOC}_{i}+\overset{˘}{\varphi }{SC}_{i}+\psi {ILOC}_{i}*{SC}_{i}+\overset{˘}{\gamma }X_{i}+\upsilon_{i}\text{,}$$

where $H_{i}$, ${SC}_{i}$, and $X_{i}$ are defined as before. We convert locus of control into a binary measure, separating those with the most external locus of control (i.e., those in the bottom quartile of the internal locus of control scale) from everybody else. Thus, *ILOC*_*i*_ equals 1 if a respondent has a locus of control score above the 25th percentile, and 0 otherwise. We use this binary measure for two reasons: First, this allows us to capture a non-linear effect, in which effects differ for people with a pronounced external locus of control compared to others—who, for simplicity, we refer to as having an internal (rather than external) locus of control, even though the level of internality may be quite heterogeneous within this group. Second, the binary measure eases interpretation of the interaction term. The threshold is, however, a somewhat arbitrary choice. Therefore, while we choose the 25th percentile as the relevant cutoff in our main estimations, we employ different thresholds such as the median or the 10th percentile in robustness checks (see below).

Our parameter of interest is ψ, which captures the interaction of an internal locus of control with increasing self-control (conditional on self-control and an internal locus of control which both also enter the equation independently). Thus, it allows us to test whether having an internal locus of control strengthens the beneficial health effects of greater self-control, indicating that people may be more likely to exercise self-control when believing it to have a positive impact.

Table 5 reports the results. An internal locus of control amplifies the beneficial effects of greater self-control in the case of all physical health outcomes as well as for health satisfaction: The interaction is significant and its direction in line with expectations, with the effect size often being at least half the size of self-control's independent effect. These results suggest a substantial amplifying effect, with a one standard deviation increase in self-control, for example, decreasing the probability of being obese by 4 percentage points (15 percent) and an additional 2 percentage points (7 percent) when combined with an internal locus of control. Interestingly, for psychological distress, the interaction term is statistically significant but operates in the opposite direction of self-control's independent effect. Still, given the coefficients' magnitudes, having an internal locus of control paired with higher self-control is still more beneficial than not having an internal locus of control. For all other mental health outcomes (i.e., MCS, mental health, and life satisfaction) the interaction is not significant, such that locus of control and self-control matter only independently of each other. Similarly, we do not find ample evidence for an internal locus of control amplifying the beneficial health effects of self-control for health behaviors either: Across most health behaviors, there is no significant interaction effect; self-control and locus of control each matter individually but not together. A key exception, however, is physical inactivity. For physical inactivity, an individual's self-control matters only when it is also coupled with an internal locus of control and not by itself, with a one standard deviation in self-control reducing physical inactivity by 1.4 percentage points (11 percent) for those who also have an internal locus of control. This is perfectly in line with our expectation that individuals' self-control only affects their take-up of physical activity when they believe being physically active has a positive impact on their lives. It is thus unsurprising that locus of control also amplifies the self-control effects for being overweight or obese—likely consequences of exercising behavior.

**Table 5:** Health outcomes regressed on internal locus of control, self-control, and their interaction.

	(1)	(2)	(3)	(4)	(5)
Panel A: Overall and Physical Health					
	Self-rated health	PCS	General health	Overweight	Obese
SC	0.131***	−0.018	4.019***	−0.042***	−0.040***
	(0.016)	(0.018)	(0.370)	(0.008)	(0.008)
	[0.039]	.	[0.061]	[-0.069]	[-0.150]
Internal LOC	0.547***	0.510***	15.467***	−0.008	−0.042***
	(0.019)	(0.021)	(0.429)	(0.010)	(0.009)
	[0.164]	.	[0.234]	[-0.012]	[-0.157]
SC*Internal LOC	0.098***	0.126***	2.167***	−0.028***	−0.020**
	(0.018)	(0.020)	(0.408)	(0.009)	(0.009)
	[0.029]	.	[0.033]	[-0.046]	[-0.074]
Adj. R^2^	0.229	0.264	0.253	0.081	0.056
Obs.	15,189	15,000	15,151	14,812	14,812
**Panel B: Health Behaviors**					
	Inactive	Smoking	Number of cigarettes	Alcohol: weekly	Alcohol: 3+ drinks
SC	−0.001	−0.059***	−2.229	−0.067***	−0.083***
	(0.007)	(0.007)	(2.102)	(0.008)	(0.009)
	[-0.007]	[-0.368]	[-0.031]	[-0.155]	[-0.173]
Internal LOC	−0.099***	−0.027***	−5.047*	0.149***	0.072***
	(0.008)	(0.007)	(3.035)	(0.009)	(0.011)
	[-0.823]	[-0.172]	[-0.071]	[0.346]	[0.148]
SC*Internal LOC	−0.014*	0.005	−0.564	0.008	0.000
	(0.008)	(0.007)	(2.488)	(0.009)	(0.010)
	[-0.113]	[0.032]	[-0.008]	[0.019]	[0.000]
Adj. R^2^	0.057	0.095	0.092	0.092	0.116
Obs.	15,234	15,178	2319	15,164	12,154
**Panel C: Mental Health and Wellbeing**					
	MCS	Mental health	Psychological distress	Life satisfaction	Health satisfaction
SC	0.267***	4.742***	−0.116***	0.227***	0.251***
	(0.019)	(0.331)	(0.007)	(0.030)	(0.038)
	.	[0.066]	[-0.580]	[0.029]	[0.035]
Internal LOC	0.970***	17.419***	−0.339***	1.020***	1.261***
	(0.021)	(0.370)	(0.009)	(0.033)	(0.044)
	.	[0.241]	[-1.697]	[0.128]	[0.176]
SC*Internal LOC	−0.024	−0.533	0.060***	0.027	0.174***
	(0.020)	(0.356)	(0.008)	(0.032)	(0.041)
	.	[-0.007]	[0.303]	[0.003]	[0.024]
Adj. R^2^	0.334	0.324	0.262	0.173	0.182
Obs.	15,000	15,233	15,256	15,283	15,281

*Notes:* HILDA wave 19, analysis sample. OLS regressions. In addition, all regressions control for gender, age (in categories), education (in categories), migrant status, Indigenous status, as well as a maximum set of fixed effects for the state of residence and a constant. Robust standard errors in parentheses. Relative effect sizes, where relevant, are in brackets. *p < 0.1, **p < 0.05, ***p < 0.01.

Again, these findings are robust to including the Big Five personality traits as additional control variables (Table A.14), to characterizing people with an internal locus of control using either their average locus of control score across waves (Table A.15) or their 2015 locus of control measure as an instrument (Table A.16), to using factor scores for locus of control and self-control (Table A.17), and to adjusting for SCQ non-response (Table A.18). We also investigate the sensitivity of our results to using the 25th percentile as a threshold for internal locus of control, estimating models using alternative thresholds. They confirm most of the patterns we find. Naturally, the internal locus of control coefficient and the interaction term are generally more pronounced when applying a more stringent definition for not having an internal locus of control by using the 10th percentile as a threshold, and weaker when splitting the population at the median locus of control (Table A.19 and A.20).

Overall, our findings thus suggest that an internal locus of control has the potential to amplify the health benefits of greater self-control—albeit not equally across all types of health outcomes. It is particularly true for all physical health outcomes, but also for physical inactivity and health satisfaction.

## 7 Conclusions

Locus of control and self-control are key personality traits for understanding people's health and health behavior. An internal locus of control and greater self-control have each been linked, for example, to a healthier lifestyle and greater physical and mental wellbeing (see, e.g., Cobb-Clark et al., 2014; Buddelmeyer & Powdthavee, 2016; Kesavayuth et al., 2020; Mofitt et al., 2011; Tangney et al., 2004; Cobb-Clark et al., 2022). Both relate to people's sense of control and are therefore sometimes used interchangeably (e.g., Fan & Jin, 2014; Stutzer & Meier, 2016). However, locus of control is people's belief about the extent of their control over what happens to them, while self-control relates to their capacity for self-regulation. The constructs are thus clearly distinct. Yet, they are conceptually related as it is only intuitive to think that a person's self-control would depend on their belief that they have some degree of influence on the course of their lives (Bandura, 1977; Rosenbaum, 1980).

In this paper we empirically study the relationship between locus of control and self-control and how they jointly predict people's health. Using population representative data and robust measures of these non-cognitive skills we make several contributions. Our results show that locus of control and self-control are distinct, moderately correlated, constructs. As expected, individuals with a more internal locus of control on average report greater self-control. Moreover, we find that self-control is indeed one mechanism through which locus of control affects health—for the first time empirically confirming Cobb-Clark's (2015) hypothesis—the extent of which, however, varies between the types of health outcomes we consider. Finally, we demonstrate that having an internal locus of control can amplify the beneficial health impacts of higher self-control, particularly for physical health, physical activity, and health satisfaction.

Our findings provide a more nuanced understanding of the behavioral underpinnings of people's health and have two important implications. First, while conceptually and empirically related, locus of control and self-control are distinct. Thus, analyses using them interchangeably in absence of measures of one or the other should be interpreted with caution. Moreover, our findings clearly indicate the benefits of including both measures in large-scale surveys for a more nuanced understanding of differences in people's behavior and outcomes. Thus, demonstrating the value of including a range of psychological constructs in surveys may set a precedent for other population representative datasets. Second, our findings underscore the importance of both internal control beliefs and greater self-control for people's physical and psychological wellbeing, making both traits excellent targets for intervention: Even though these personality traits are rather stable in adulthood, there is some evidence that training can be effective (see Friese et al., 2017, for a meta-study on self-control) and they have been shown to be malleable especially during childhood with several intervention programs proven to be successful in different contexts (see Piquero et al., 2010, 2016, for self-control; Craig et al., 1998, Wolinsky et al., 2010, Burgoyne et al., 2018; Galvin et al., 2018, for locus of control). Early intervention may thereby yield long-term benefits. Moreover, while self-control appears to be one channel through which locus of control improves health outcomes, the two are also often reinforcing: An internal locus of control can amplify the beneficial health effects of greater self-control. This implies that there could be great efficiency gains by targeting both locus of control and self-control simultaneously.

While our focus in this paper is on physical and mental health as well as health behaviors, there is no a priori reason to believe that our findings are limited to people's health and wellbeing. Independently, both locus of control and self-control have also been linked to more favorable outcomes and behaviors across a range of other domains, including finances, education, and labor market outcomes (see, e.g., Caliendo et al., 2015; Cobb-Clark, 2015; Cobb-Clark et al., 2022; Duckworth & Seligman, 2005). Therefore, future research that investigates whether our results extend to other domains of people's life would be valuable. People's labor market outcomes—which have been demonstrated to be affected by both locus of control and self-control as well—would be a great place to start. Potential interactions between an internal locus of control and greater self-control would be particularly informative for improving the targeting of labor market policies directed, for example, at the long-term unemployed. They would also greatly raise the societal returns to behavioral intervention, with health policies targeting locus of control and self-control having positive spillover effects into other domains.

## Conflict of interest

None.

## Ethical statement

This paper uses unit record data from Household, Income and Labour Dynamics in Australia Survey [HILDA] conducted by the Australian Government Department of Social Services (DSS). The findings and views reported in this paper, however, are those of the authors and should not be attributed to the Australian Government, DSS, or any of DSS’ contractors or partners. DOI: 10.26193/YP7MNU.

## CRediT authorship contribution statement

**Ferdi Botha:** Conceptualization, Formal analysis, Funding acquisition, Methodology, Project administration, Writing – original draft, Writing – review & editing. **Sarah C. Dahmann:** Conceptualization, Formal analysis, Funding acquisition, Methodology, Project administration, Writing – original draft, Writing – review & editing.

## Declaration of competing interest

Declarations of interests: None.

## Data Availability

The authors do not have permission to share data.
