# Supplementary material for: Locus of control, self-control, and health outcomes
Source: SSM Popul Health. 2023 Nov 24;25:101566. doi: 10.1016/j.ssmph.2023.101566 (PMC10698268; doi:10.1016/j.ssmph.2023.101566)
Supplement: Multimedia component 1 [file mmc1.docx]

**SUPPLEMENTARY DATA**

**APPENDIX A**

**Table A.1: Pearlin and Schooler’s (1978) Mastery Scale to measure Locus of Control**

| Item | Question |
| --- | --- |
| 1 | I have little control over the things that happen to me. *[reversed]* |
| 2 | There is really no way I can solve some of the problems I have. *[reversed]* |
| 3 | There is little I can do to change many of the important things in my life. *[reversed]* |
| 4 | I often feel helpless in dealing with the problems of life. *[reversed]* |
| 5 | Sometimes I feel that I’m being pushed around in life. *[reversed]* |
| 6 | What happens to me in the future mostly depends on me. |
| 7 | I can do just about anything I really set my mind to do. |

*Notes:* Respondents are asked much they agree with the statements, with responses to each question range from 1 (“strongly disagree”) to 7 (“strongly agree”). We reverse responses to items marked as “[reversed]”.

**Table A.2: Brief Self-Control Scale (Tangney et al., 2004) to measure Self-Control**

| Item | Question |
| --- | --- |
| 1 | I am good at resisting temptation. |
| 2 | I have a hard time breaking bad habits. *[reversed]* |
| 3 | I am lazy. *[reversed]* |
| 4 | I say inappropriate things. *[reversed]* |
| 5 | I do certain things that are bad for me, if they are fun. *[reversed]* |
| 6 | I refuse things that are bad for me. |
| 7 | I wish I had more self-discipline. *[reversed]* |
| 8 | People would say I have iron self-discipline. |
| 9 | Pleasure and fun sometimes keep me from getting work done. *[reversed]* |
| 10 | I have trouble concentrating. *[reversed]* |
| 11 | I can work effectively towards long-term goals. |
| 12 | Sometimes I cannot stop myself from doing something even if I know it is wrong. *[reversed]* |
| 13 | I often act without thinking through all the alternatives. *[reversed]* |

*Notes:* Respondents are asked to rate how well each statement describes them, with responses ranging from 1 (“not at all”) to 5 (“very well”). We reverse responses to items marked as “[reversed]”.

**Table A.3: Reliability of Locus of Control scale**

| Item | Sign | Item-test correlation | Item-rest correlation | Average interitem covariance | Cronbach’s Alpha |
| --- | --- | --- | --- | --- | --- |
| LOC1 | + | 0.7299 | 0.6094 | 1.102642 | 0.8182 |
| LOC2 | + | 0.8004 | 0.7060 | 1.043020 | 0.8027 |
| LOC3 | + | 0.8025 | 0.7117 | 1.049469 | 0.8022 |
| LOC4 | + | 0.8025 | 0.7106 | 1.046066 | 0.8022 |
| LOC5 | + | 0.7472 | 0.6344 | 1.091573 | 0.8142 |
| LOC6 | + | 0.5122 | 0.3433 | 1.299644 | 0.8570 |
| LOC7 | + | 0.6136 | 0.4728 | 1.220191 | 0.8380 |
| Test scale |  |  |  | 1.121801 | 0.8419 |
| *Notes:* HILDA wave 19, analysis sample with 15,288 observations. | | | | | |

**Table A.4: Reliability of Brief Self-Control Scale**

| Item | Sign | Item-test correlation | Item-rest correlation | Average interitem covariance | Cronbach’s Alpha |
| --- | --- | --- | --- | --- | --- |
| BSCS1 | + | 0.6091 | 0.5203 | 0.357375 | 0.8213 |
| BSCS2 | + | 0.5828 | 0.4870 | 0.359566 | 0.8235 |
| BSCS3 | + | 0.5826 | 0.4887 | 0.360518 | 0.8234 |
| BSCS4 | + | 0.5591 | 0.4595 | 0.362807 | 0.8254 |
| BSCS5 | + | 0.6510 | 0.5598 | 0.346289 | 0.8180 |
| BSCS6 | + | 0.5064 | 0.3912 | 0.367863 | 0.8306 |
| BSCS7 | + | 0.6522 | 0.5538 | 0.342103 | 0.8183 |
| BSCS8 | + | 0.4974 | 0.3854 | 0.370441 | 0.8308 |
| BSCS9 | + | 0.5605 | 0.4554 | 0.360474 | 0.8258 |
| BSCS10 | + | 0.6071 | 0.5113 | 0.354375 | 0.8217 |
| BSCS11 | + | 0.4719 | 0.3713 | 0.377975 | 0.8310 |
| BSCS12 | + | 0.6494 | 0.5615 | 0.348551 | 0.8181 |
| BSCS13 | + | 0.6017 | 0.5127 | 0.358820 | 0.8218 |
| Test scale |  |  |  | 0.359012 | 0.8352 |
| *Notes:* HILDA wave 19, analysis sample with 15,288 observations. | | | | | |

**Table A.5: Variable definitions**

| Variable | Definition |
| --- | --- |
| Locus of control | Pearlin and Schooler’s (1978) 7-item Mastery Scale. |
| Self-control | 13-item Brief Self-Control Scale by Tangney et al. (2004). |
| **Health outcomes** |  |
| *Overall and physical health* |  |
| Self-rated health | Responses to the question “In general, would you say your health is ‘excellent’, ‘very good’, ‘good’, ‘fair’, ‘poor’. Measured on 5-point Likert scale ranging from 1 (poor) to 5 (excellent). |
| PCS | Physical Health Component Summary Score derived from the SF-36 measure. The PCS is obtained from principal components analysis that identifies the physical functioning, role-physical, bodily pain, and general health components as part of the physical health dimension. The final score is standardized with mean of 0 and standard deviation of 1. |
| General health | General Health subscale of the SF-36 measure, ranging from 0 (low) to 100 (high). |
| Overweight | = 1 if a respondent has a Body Mass Index (body weight in kg divided body height in meter squared) greater than or equal to 25, 0 otherwise. |
| Obese | = 1 if a respondent has a Body Mass Index (body weight in kg divided body height in meter squared) greater than or equal to 30, 0 otherwise. |
| *Health behaviors* |  |
| Inactive | Responses to the question “In general, how often do you participate in moderate or intensive physical activity for at least 30 minutes?” Variable = 1 if response is “not at all”, 0 otherwise. |
| Smoking | = 1 if respondent is currently a smoker, 0 otherwise. |
| Number of cigarettes | Number of cigarettes usually smoked per week, if a smoker. |
| Alcohol: weekly | = 1 if respondent reports drinking alcohol at least once a week, 0 otherwise. |
| Alcohol: 3+ drinks | = 1 if respondent reports drinking at least three standard drinks per occasion, 0 otherwise. |
| *Mental health and wellbeing* |  |
| MCS | Mental Health Component Summary Score derived from the SF-36 measure. The MCS is obtained from principal components analysis that identifies the vitality, social functioning, role-emotional, and mental health components as part of the mental health dimension. The final score is standardized with mean of 0 and standard deviation of 1. |
| Mental health | Mental Health subscale of the SF-36 measure, ranging from 0 (low) to 100 (high). |
| Psychological distress | = 1 if a respondent’s Kessler-10 score on a scale of 10-50 is equal to or greater than 30 (indicating high or very high distress), and 0 otherwise. |
| Life satisfaction | Response to the question “All things considered, how satisfied are you with your life?” Measured on an 11-point Likert scale ranging from 0 (very dissatisfied) to 10 (very satisfied). |
| Health satisfaction | Response to the question “All things considered, how satisfied are you with your health?” Measured on an 11-point Likert scale ranging from 0 (very dissatisfied) to 10 (very satisfied). |
| **Control variables** |  |
| Male | = 1 if a respondent is male, 0 otherwise. |
| Age | Respondent’s age in 2019 in brackets, 15-24, 25-34. 35-44, 45-54, 55-64, 65 and over. |
| Education | Respondent’s highest level of education achieved. (i) Year 11 and below, (ii) Year 12, (iii) Certificate III or IV, or Advanced Diploma, (vi) Bachelor degree or higher. |
| Migrant status | Respondent’s country of birth. Australian-born, Migrant from main English-speaking country, Migrant from country other than the main English-speaking countries. |
| Indigenous | = 1 if a respondent identifies as Indigenous or Torres Strait Islander, 0 otherwise. |
| State | State of residence of respondent. Includes New South Wales, Victoria, Queensland, South Australia, Western Australia, Tasmania, Northern Territory, Australian Capital Territory. |

**Table A.6: Summary statistics**

|  | Obs. | Mean | Std. dev. | Min. | Max. |
| --- | --- | --- | --- | --- | --- |
| **Locus of control (LOC) and self-control (SC)** | | | | | |
| LOC | 15,288 | 0.000 | 1.000 | -3.82 | 1.38 |
| SC | 15,288 | 0.000 | 1.000 | -3.76 | 2.34 |
| **Health outcomes** |  |  |  |  |  |
| Self-rated health | 15,189 | 3.344 | 0.968 | 1 | 5 |
| PCS | 15,000 | 0.000 | 1.000 | -4.19 | 1.97 |
| General health | 15,151 | 66.215 | 21.105 | 0 | 100 |
| Overweight | 14,812 | 0.609 | 0.488 | 0 | 1 |
| Obese | 14,812 | 0.268 | 0.443 | 0 | 1 |
| Inactive | 15,234 | 0.120 | 0.325 | 0 | 1 |
| Smoking | 15,178 | 0.160 | 0.366 | 0 | 1 |
| Number of cigarettes | 2,319 | 71.129 | 59.562 | 1 | 400 |
| Alcohol: weekly | 15,164 | 0.431 | 0.495 | 0 | 1 |
| Alcohol: 3+ drinks | 12,154 | 0.483 | 0.500 | 0 | 1 |
| MCS | 15,000 | 0.000 | 1.000 | -4.27 | 2.17 |
| Mental health | 15,233 | 72.361 | 18.149 | 0 | 100 |
| Psychological distress | 15,256 | 0.200 | 0.400 | 0 | 1 |
| Life satisfaction | 15,283 | 7.972 | 1.415 | 0 | 10 |
| Health satisfaction | 15,281 | 7.174 | 1.926 | 0 | 10 |
| **Control variables** |  |  |  |  |  |
| Male | 15,288 | 0.472 | 0.499 | 0 | 1 |
| *Age* |  |  |  |  |  |
| 15-24 | 15,288 | 0.147 | 0.354 | 0 | 1 |
| 25-34 | 15,288 | 0.195 | 0.396 | 0 | 1 |
| 35-44 | 15,288 | 0.149 | 0.356 | 0 | 1 |
| 45-54 | 15,288 | 0.152 | 0.359 | 0 | 1 |
| 55-64 | 15,288 | 0.157 | 0.364 | 0 | 1 |
| 65+ | 15,288 | 0.200 | 0.400 | 0 | 1 |
| *Education* |  |  |  |  |  |
| Year 11 and below | 15,288 | 0.229 | 0.421 | 0 | 1 |
| Year 12 | 15,288 | 0.152 | 0.359 | 0 | 1 |
| Cert III or IV, or (Adv.) Diploma | 15,288 | 0.332 | 0.471 | 0 | 1 |
| Bachelor degree or higher | 15,288 | 0.286 | 0.452 | 0 | 1 |
| *Migrant status* |  |  |  |  |  |
| Australian | 15,288 | 0.801 | 0.399 | 0 | 1 |
| Main English speaking | 15,288 | 0.089 | 0.285 | 0 | 1 |
| Other migrant | 15,288 | 0.110 | 0.312 | 0 | 1 |
| Indigenous | 15,288 | 0.030 | 0.170 | 0 | 1 |
| *State* |  |  |  |  |  |
| NSW | 15,288 | 0.285 | 0.452 | 0 | 1 |
| VIC | 15,288 | 0.254 | 0.435 | 0 | 1 |
| QLD | 15,288 | 0.219 | 0.414 | 0 | 1 |
| SA | 15,288 | 0.089 | 0.284 | 0 | 1 |
| WA | 15,288 | 0.089 | 0.284 | 0 | 1 |
| TAS | 15,288 | 0.034 | 0.182 | 0 | 1 |
| NT | 15,288 | 0.008 | 0.089 | 0 | 1 |
| ACT | 15,288 | 0.022 | 0.147 | 0 | 1 |
| *Notes:* HILDA wave 19, analysis sample. See Table A.5 for variable definitions. | | | | | |

**Table A.7: Full sample and analysis sample comparisons**

|  | Full sample | |  | Analysis sample | |  | Equality of means | |
| --- | --- | --- | --- | --- | --- | --- | --- | --- |
|  | Mean | Obs. |  | Mean | Obs. |  | t-stat | p-value |
| Locus of control | 0.000 | 15,774 |  | 0.007 | 15,288 |  | -0.579 | 0.563 |
| Self-control | 0.000 | 15,463 |  | 0.002 | 15,288 |  | -0.172 | 0.864 |
| Self-rated health | 3.333 | 15,969 |  | 3.344 | 15,189 |  | -0.941 | 0.347 |
| PCS | 0.000 | 15,698 |  | 0.017 | 15,000 |  | -1.505 | 0.132 |
| General health | 65.974 | 15,886 |  | 66.215 | 15,151 |  | -1.002 | 0.316 |
| Overweight | 0.609 | 15,478 |  | 0.609 | 14,812 |  | 0.066 | 0.948 |
| Obese | 0.270 | 15,478 |  | 0.268 | 14,812 |  | 0.256 | 0.798 |
| Inactive | 0.124 | 15,989 |  | 0.120 | 15,234 |  | 0.968 | 0.333 |
| Smoking | 0.162 | 15,918 |  | 0.160 | 15,178 |  | 0.587 | 0.557 |
| Number of cigarettes | 70.571 | 2,464 |  | 71.129 | 2,319 |  | -0.324 | 0.746 |
| Alcohol: weekly | 0.426 | 15,911 |  | 0.431 | 15,164 |  | -0.793 | 0.428 |
| Alcohol: 3+ drinks | 0.482 | 12,650 |  | 0.483 | 12,154 |  | -0.094 | 0.925 |
| MCS | 0.000 | 15,698 |  | 0.007 | 15,000 |  | -0.576 | 0.565 |
| Mental health | 72.182 | 15,987 |  | 72.361 | 15,233 |  | -0.870 | 0.385 |
| Psychological distress | 0.203 | 15,992 |  | 0.200 | 15,256 |  | 0.072 | 0.440 |
| Life satisfaction | 7.952 | 17,449 |  | 7.972 | 15,283 |  | -1.260 | 0.208 |
| Health satisfaction | 7.161 | 17,446 |  | 7.174 | 15,281 |  | -0.562 | 0.574 |
| Male | 0.474 | 17,462 |  | 0.472 | 15,288 |  | 0.450 | 0.653 |
| Age: 15-24 | 0.150 | 17,462 |  | 0.147 | 15,288 |  | 0.734 | 0.463 |
| Age: 25-34 | 0.200 | 17,462 |  | 0.195 | 15,288 |  | 1.057 | 0.290 |
| Age: 35-44 | 0.151 | 17,462 |  | 0.149 | 15,288 |  | 0.481 | 0.631 |
| Age: 45-54 | 0.149 | 17,462 |  | 0.152 | 15,288 |  | -0.779 | 0.436 |
| Age: 55-64 | 0.151 | 17,462 |  | 0.157 | 15,288 |  | -1.692 | 0.091 |
| Age: 65+ | 0.200 | 17,462 |  | 0.200 | 15,288 |  | 0.089 | 0.929 |
| Education: Year 11 and below | 0.242 | 17,453 |  | 0.229 | 15,288 |  | 2.718 | 0.007 |
| Education: Year 12 | 0.152 | 17,453 |  | 0.152 | 15,288 |  | -0.059 | 0.953 |
| Education: Cert III/IV/Diploma | 0.330 | 17,453 |  | 0.332 | 15,288 |  | -0.231 | 0.818 |
| Education: Bachelor degree/higher | 0.275 | 17,453 |  | 0.286 | 15,288 |  | -2.282 | 0.023 |
| Country of birth: Australian | 0.798 | 17,458 |  | 0.801 | 15,288 |  | -0.625 | 0.532 |
| Country of birth: Main English speaking | 0.087 | 17,458 |  | 0.089 | 15,288 |  | -0.654 | 0.513 |
| Country of birth: Other migrant | 0.114 | 17,458 |  | 0.110 | 15,288 |  | 1.381 | 0.167 |
| Indigenous | 0.034 | 17,462 |  | 0.030 | 15,288 |  | 2.056 | 0.040 |
| State: NSW | 0.289 | 17,462 |  | 0.285 | 15,288 |  | 0.637 | 0.524 |
| State: VIC | 0.254 | 17,462 |  | 0.254 | 15,288 |  | 0.090 | 0.929 |
| State: QLD | 0.218 | 17,462 |  | 0.219 | 15,288 |  | -0.157 | 0.876 |
| State: SA | 0.089 | 17,462 |  | 0.089 | 15,288 |  | 0.117 | 0.907 |
| State: WA | 0.088 | 17,462 |  | 0.089 | 15,288 |  | -0.273 | 0.785 |
| State: TAS | 0.033 | 17,462 |  | 0.034 | 15,288 |  | -0.588 | 0.556 |
| State: NT | 0.008 | 17,462 |  | 0.008 | 15,288 |  | -0.028 | 0.977 |
| State: ACT | 0.021 | 17,462 |  | 0.022 | 15,288 |  | -0.755 | 0.450 |
| *Notes:* HILDA wave 19, full sample and analysis sample. See Table A.5 for variable definitions. | | | | | | | | |

**Table A.8: Sensitivity tests for self-control regressed on locus of control**

|  | (1) | (2) |
| --- | --- | --- |
| LOC | 0.337*** | 0.364*** |
|  | (0.012) | (0.008) |
| Adj. R^2^ | 0.117 | 0.132 |
| Obs. | 15,088 | 15,288 |
| *Notes:* HILDA wave 19, analysis sample. OLS regressions with self-control as outcome variable. Column (1) is a weighted regression, using self-completion questionnaire weights. In column (2) locus of control and self-control are constructed as factor scores derived from two separate factor analyses. In addition, specifications in all columns control for a constant. Robust standard errors in parentheses. * p<0.1, ** p<0.05, *** p<0.01. | | |

**Table A.9: Health outcomes regressed on locus of control and self-control, controlling for Big Five personality traits**

|  | (1) | (2) | (3) | (4) | (5) | (6) | (7) | (8) | (9) | (10) |
| --- | --- | --- | --- | --- | --- | --- | --- | --- | --- | --- |
| **Panel A: Overall and Physical Health** | | | | | | | | | | |
|  | Self-rated health | | PCS | | General health | | Overweight | | Obese | |
| LOC | 0.312*** | 0.281*** | 0.268*** | 0.258*** | 8.849*** | 7.993*** | -0.009** | 0.004 | -0.027*** | -0.015*** |
|  | (0.009) | (0.009) | (0.009) | (0.010) | (0.189) | (0.195) | (0.005) | (0.005) | (0.004) | (0.004) |
|  | [0.093] | [0.084] | . | . | [0.134] | [0.121] | [-0.015] | [0.007] | [-0.102] | [-0.057] |
| SC |  | 0.136*** |  | 0.042*** |  | 3.790*** |  | -0.060*** |  | -0.054*** |
|  |  | (0.009) |  | (0.010) |  | (0.205) |  | (0.005) |  | (0.005) |
|  |  | [0.041] |  | . |  | [0.057] |  | [-0.098] |  | [-0.200] |
| $\chi^{2}$ test | . | 157.7*** | . | 18.7*** | . | 227.8*** | . | 109.5*** | . | 106.1*** |
| Adj. R^2^ | 0.248 | 0.261 | 0.278 | 0.279 | 0.285 | 0.305 | 0.065 | 0.075 | 0.052 | 0.061 |
| Obs. | 13,279 | 13,279 | 13,120 | 13,120 | 13,240 | 13,240 | 12,983 | 12,983 | 12,983 | 12,983 |
| **Panel B: Health Behaviors** | | | | | | | | | | |
|  | Inactive | | Smoking | | Number of cigarettes | | Alcohol: weekly | | Alcohol: 3+ drinks | |
| LOC | -0.054*** | -0.052*** | -0.020*** | -0.008** | -2.407* | -1.879 | 0.055*** | 0.072*** | 0.019*** | 0.040*** |
|  | (0.004) | (0.004) | (0.003) | (0.004) | (1.387) | (1.402) | (0.005) | (0.005) | (0.005) | (0.005) |
|  | [-0.446] | [-0.437] | [-0.126] | [-0.050] | [-0.034] | [-0.026] | [0.128] | [0.166] | [0.040] | [0.082] |
| SC |  | -0.005 |  | -0.054*** |  | -2.565* |  | -0.073*** |  | -0.091*** |
|  |  | (0.004) |  | (0.004) |  | (1.548) |  | (0.005) |  | (0.006) |
|  |  | [-0.040] |  | [-0.337] |  | [-0.036] |  | [-0.170] |  | [-0.189] |
| $\chi^{2}$ test | . | 1.7 | . | 152.3*** | . | 2.7* | . | 156.9*** | . | 179.5*** |
| Adj. R^2^ | 0.062 | 0.062 | 0.083 | 0.097 | 0.091 | 0.091 | 0.072 | 0.086 | 0.108 | 0.129 |
| Obs. | 13,315 | 13,315 | 13,269 | 13,269 | 1,902 | 1,902 | 13,252 | 13,252 | 10,867 | 10,867 |
| **Panel C: Mental Health and Wellbeing** | | | | | | | | | | |
|  | MCS | | Mental health | | Psychological distress | | Life satisfaction | | Health satisfaction | |
| LOC | 0.519*** | 0.487*** | 9.253*** | 8.742*** | -0.165*** | -0.158*** | 0.553*** | 0.528*** | 0.689*** | 0.639*** |
|  | (0.008) | (0.008) | (0.152) | (0.156) | (0.004) | (0.004) | (0.015) | (0.015) | (0.019) | (0.020) |
|  | . | . | [0.128] | [0.121] | [-0.828] | [-0.790] | [0.069] | [0.066] | [0.096] | [0.089] |
| SC |  | 0.142*** |  | 2.258*** |  | -0.034*** |  | 0.112*** |  | 0.223*** |
|  |  | (0.009) |  | (0.159) |  | (0.004) |  | (0.014) |  | (0.019) |
|  |  | . |  | [0.031] |  | [-0.170] |  | [0.014] |  | [0.031] |
| $\chi^{2}$ test | . | 181.2*** | . | 154.0*** | . | 77.8*** | . | 58.4*** | . | 110.9*** |
| Adj. R^2^ | 0.405 | 0.418 | 0.403 | 0.413 | 0.278 | 0.283 | 0.238 | 0.242 | 0.213 | 0.221 |
| Obs. | 13,120 | 13,120 | 13,314 | 13,314 | 13,333 | 13,333 | 13,357 | 13,357 | 13,354 | 13,354 |
| *Notes:* HILDA wave 19, analysis sample. OLS regressions. All regressions control for gender, age (in categories), education (in categories), migrant status, Indigenous status, standardized measures of each Big Five personality trait (openness to new experiences, conscientiousness, extraversion, agreeableness, emotional stability), state of residence fixed effects and a constant. Robust standard errors in parentheses. Relative effect sizes, where relevant, are in brackets. $\chi^{2}$ test is a test of the null hypothesis that the locus of control coefficients from the initial (without self-control) and subsequent (with self-control) model are not statistically different from each other. * p<0.1, ** p<0.05, *** p<0.01. | | | | | | | | | | |

**Table A.10: Health outcomes regressed on average locus of control and self-control**

|  | (1) | (2) | (3) | (4) | (5) | (6) | (7) | (8) | (9) | (10) |
| --- | --- | --- | --- | --- | --- | --- | --- | --- | --- | --- |
| **Panel A: Overall and Physical Health** | | | | | | | | | | |
|  | Self-rated health | | PCS | | General health | | Overweight | | Obese | |
| LOC | 0.333*** | 0.271*** | 0.250*** | 0.232*** | 9.582*** | 7.898*** | -0.019*** | 0.005 | -0.036*** | -0.016*** |
|  | (0.007) | (0.008) | (0.008) | (0.009) | (0.163) | (0.178) | (0.004) | (0.004) | (0.004) | (0.004) |
|  | [0.100] | [0.081] | . | . | [0.145] | [0.119] | [-0.031] | [0.007] | [-0.133] | [-0.060] |
| SC |  | 0.168*** |  | 0.050*** |  | 4.543*** |  | -0.065*** |  | -0.054*** |
|  |  | (0.008) |  | (0.008) |  | (0.175) |  | (0.004) |  | (0.004) |
|  |  | [0.050] |  | . |  | [0.069] |  | [-0.107] |  | [-0.200] |
| $\chi^{2}$ test | . | 364.1*** | . | 36.7*** | . | 518.4*** | . | 199.1*** | . | 168.1*** |
| Adj. R^2^ | 0.219 | 0.243 | 0.266 | 0.268 | 0.246 | 0.283 | 0.067 | 0.081 | 0.044 | 0.056 |
| Obs. | 15,189 | 15,189 | 15,000 | 15,000 | 15,151 | 15,151 | 14,812 | 14,812 | 14,812 | 14,812 |
| **Panel B: Health Behaviors** | | | | | | | | | | |
|  | Inactive | | Smoking | | Number of cigarettes | | Alcohol: weekly | | Alcohol: 3+ drinks | |
| LOC | -0.044*** | -0.041*** | -0.036*** | -0.016*** | -2.622** | -1.854 | 0.049*** | 0.076*** | 0.007 | 0.040*** |
|  | (0.003) | (0.003) | (0.003) | (0.003) | (1.202) | (1.284) | (0.004) | (0.004) | (0.004) | (0.005) |
|  | [-0.366] | [-0.343] | [-0.223] | [-0.101] | [-0.037] | [-0.026] | [0.115] | [0.176] | [0.014] | [0.083] |
| SC |  | -0.007** |  | -0.052*** |  | -2.563** |  | -0.071*** |  | -0.090*** |
|  |  | (0.003) |  | (0.003) |  | (1.296) |  | (0.004) |  | (0.005) |
|  |  | [-0.062] |  | [-0.329] |  | [-0.036] |  | [-0.164] |  | [-0.185] |
| $\chi^{2}$ test | . | 6.1** | . | 229.8*** | . | 3.9** | . | 242.9*** | . | 293.7*** |
| Adj. R^2^ | 0.055 | 0.055 | 0.080 | 0.096 | 0.090 | 0.091 | 0.080 | 0.096 | 0.093 | 0.118 |
| Obs. | 15,234 | 15,234 | 15,178 | 15,178 | 2,319 | 2,319 | 15,164 | 15,164 | 12,154 | 12,154 |
| **Panel C: Mental Health and Wellbeing** | | | | | | | | | | |
|  | MCS | | Mental health | | Psychological distress | | Life satisfaction | | Health satisfaction | |
| LOC | 0.548*** | 0.476*** | 9.871*** | 8.641*** | -0.183*** | -0.163*** | 0.609*** | 0.547*** | 0.734*** | 0.623*** |
|  | (0.007) | (0.008) | (0.135) | (0.148) | (0.003) | (0.003) | (0.013) | (0.014) | (0.016) | (0.018) |
|  | . | . | [0.136] | [0.119] | [-0.916] | [-0.815] | [0.076] | [0.069] | [0.102] | [0.087] |
| SC |  | 0.194*** |  | 3.320*** |  | -0.055*** |  | 0.170*** |  | 0.300*** |
|  |  | (0.008) |  | (0.144) |  | (0.003) |  | (0.012) |  | (0.017) |
|  |  | . |  | [0.046] |  | [-0.274] |  | [0.021] |  | [0.042] |
| $\chi^{2}$ test | . | 442.6*** | . | 420.9*** | . | 247.2*** | . | 177.9*** | . | 277.6*** |
| Adj. R^2^ | 0.328 | 0.357 | 0.324 | 0.350 | 0.242 | 0.257 | 0.198 | 0.209 | 0.181 | 0.200 |
| Obs. | 15,000 | 15,000 | 15,233 | 15,233 | 15,256 | 15,256 | 15,283 | 15,283 | 15,281 | 15,281 |
| *Notes:* HILDA wave 19, analysis sample. OLS regressions. LOC is constructed here as the average locus of control score for all relevant waves an individual is observed in the HILDA longitudinal file. All regressions control for gender, age (in categories), education (in categories), migrant status, Indigenous status, state of residence fixed effects and a constant. Robust standard errors in parentheses. Relative effect sizes, where relevant, are in brackets. $\chi^{2}$ test is a test of the null hypothesis that the locus of control coefficients from the initial (without self-control) and subsequent (with self-control) model are not statistically different from each other. * p<0.1, ** p<0.05, *** p<0.01. | | | | | | | | | | |

**Table A.11: Health outcomes regressed on locus of control (instrumented) and self-control**

|  | (1) | (2) | (3) | (4) | (5) | (6) | (7) | (8) | (9) | (10) |
| --- | --- | --- | --- | --- | --- | --- | --- | --- | --- | --- |
| **Panel A: Overall and Physical Health** | | | | | | | | | | |
|  | Self-rated health | | PCS | | General health | | Overweight | | Obese | |
| LOC | 0.485*** | 0.429*** | 0.397*** | 0.399*** | 13.879*** | 12.456*** | -0.033*** | 0.000 | -0.058*** | -0.032*** |
|  | (0.014) | (0.017) | (0.016) | (0.019) | (0.319) | (0.384) | (0.008) | (0.009) | (0.007) | (0.009) |
|  | [0.145] | [0.128] | . | . | [0.210] | [0.188] | [-0.054] | [0.001] | [-0.217] | [-0.119] |
| SC |  | 0.108*** |  | -0.004 |  | 2.744*** |  | -0.064*** |  | -0.051*** |
|  |  | (0.011) |  | (0.012) |  | (0.244) |  | (0.006) |  | (0.005) |
|  |  | [0.032] |  | . |  | [0.041] |  | [-0.106] |  | [-0.189] |
| Adj. R^2^ | 0.222 | 0.244 | 0.261 | 0.261 | 0.241 | 0.275 | 0.047 | 0.062 | 0.038 | 0.052 |
| Obs. | 11,931 | 11,931 | 11,789 | 11,789 | 11,897 | 11,897 | 11,689 | 11,689 | 11,689 | 11,689 |
| **Panel B: Health Behaviors** | | | | | | | | | | |
|  | Inactive | | Smoking | | Number of cigarettes | | Alcohol: weekly | | Alcohol: 3+ drinks | |
| LOC | -0.063*** | -0.064*** | -0.053*** | -0.030*** | -7.047** | -6.933** | 0.088*** | 0.137*** | 0.011 | 0.065*** |
|  | (0.006) | (0.007) | (0.006) | (0.007) | (2.838) | (3.270) | (0.008) | (0.009) | (0.009) | (0.011) |
|  | [-0.525] | [-0.535] | [-0.331] | [-0.190] | [-0.099] | [-0.097] | [0.205] | [0.317] | [0.022] | [0.135] |
| SC |  | 0.003 |  | -0.043*** |  | -0.298 |  | -0.094*** |  | -0.101*** |
|  |  | (0.004) |  | (0.004) |  | (1.917) |  | (0.006) |  | (0.006) |
|  |  | [0.021] |  | [-0.272] |  | [-0.004] |  | [-0.217] |  | [-0.208] |
| Adj. R^2^ | 0.057 | 0.057 | 0.067 | 0.087 | 0.071 | 0.071 | 0.052 | 0.061 | 0.088 | 0.113 |
| Obs. | 11,967 | 11,967 | 11,926 | 11,926 | 1,701 | 1,701 | 11,905 | 11,905 | 9,789 | 9,789 |
| **Panel C: Mental Health and Wellbeing** | | | | | | | | | | |
|  | MCS | | Mental health | | Psychological distress | | Life satisfaction | | Health satisfaction | |
| LOC | 0.731*** | 0.678*** | 13.271*** | 12.412*** | -0.235*** | -0.225*** | 0.865*** | 0.846*** | 1.077*** | 1.002*** |
|  | (0.014) | (0.017) | (0.261) | (0.313) | (0.006) | (0.007) | (0.024) | (0.028) | (0.031) | (0.038) |
|  | . | . | [0.183] | [0.172] | [-1.178] | [-1.124] | [0.108] | [0.106] | [0.150] | [0.140] |
| SC |  | 0.102*** |  | 1.657*** |  | -0.021*** |  | 0.037** |  | 0.145*** |
|  |  | (0.011) |  | (0.192) |  | (0.004) |  | (0.016) |  | (0.023) |
|  |  | . |  | [0.023] |  | [-0.103] |  | [0.005] |  | [0.020] |
| Adj. R^2^ | 0.357 | 0.380 | 0.351 | 0.371 | 0.241 | 0.250 | 0.201 | 0.206 | 0.179 | 0.194 |
| Obs. | 11,789 | 11,789 | 11,967 | 11,967 | 11,976 | 11,976 | 11,998 | 11,998 | 11,998 | 11,998 |
| *Notes:* HILDA wave 19, analysis sample. 2SLS regressions. LOC is an individual’s 2019 locus of control score, which is instrumented by the individual’s 2015 locus of control score. All regressions control for gender, age (in categories), education (in categories), migrant status, Indigenous status, state of residence fixed effects and a constant. Robust standard errors in parentheses. Relative effect sizes, where relevant, are in brackets. * p<0.1, ** p<0.05, *** p<0.01. | | | | | | | | | | |

**Table A.12: Health outcomes regressed on locus of control and self-control, using factor scores for locus of control and self-control**

|  | (1) | (2) | (3) | (4) | (5) | (6) | (7) | (8) | (9) | (10) |
| --- | --- | --- | --- | --- | --- | --- | --- | --- | --- | --- |
| **Panel A: Overall and Physical Health** | | | | | | | | | | |
|  | Self-rated health | | PCS | | General health | | Overweight | | Obese | |
| LOC | 0.350*** | 0.293*** | 0.260*** | 0.245*** | 9.916*** | 8.349*** | -0.018*** | 0.005 | -0.035*** | -0.016*** |
|  | (0.007) | (0.008) | (0.008) | (0.009) | (0.164) | (0.179) | (0.004) | (0.004) | (0.004) | (0.004) |
|  | [0.105] | [0.088] | . | . | [0.150] | [0.126] | [-0.030] | [0.009] | [-0.131] | [-0.060] |
| SC |  | 0.154*** |  | 0.042*** |  | 4.235*** |  | -0.064*** |  | -0.052*** |
|  |  | (0.008) |  | (0.008) |  | (0.173) |  | (0.004) |  | (0.004) |
|  |  | [0.046] |  | . |  | [0.064] |  | [-0.105] |  | [-0.193] |
| $\chi^{2}$ test | . | 317.9*** | . | 26.1*** | . | 469.7*** | . | 190.1*** | . | 154.9*** |
| Adj. R^2^ | 0.233 | 0.253 | 0.272 | 0.273 | 0.264 | 0.295 | 0.067 | 0.080 | 0.044 | 0.055 |
| Obs. | 15,189 | 15,189 | 15,000 | 15,000 | 15,151 | 15,151 | 14,812 | 14,812 | 14,812 | 14,812 |
| **Panel B: Health Behaviors** | | | | | | | | | | |
|  | Inactive | | Smoking | | Number of cigarettes | | Alcohol: weekly | | Alcohol: 3+ drinks | |
| LOC | -0.051*** | -0.050*** | -0.031*** | -0.011*** | -2.410** | -1.593 | 0.048*** | 0.074*** | 0.006 | 0.039*** |
|  | (0.003) | (0.003) | (0.003) | (0.003) | (1.159) | (1.216) | (0.004) | (0.004) | (0.004) | (0.005) |
|  | [-0.428] | [-0.420] | [-0.193] | [-0.067] | [-0.034] | [-0.022] | [0.111] | [0.173] | [0.012] | [0.081] |
| SC |  | -0.002 |  | -0.054*** |  | -2.613** |  | -0.072*** |  | -0.090*** |
|  |  | (0.003) |  | (0.003) |  | (1.257) |  | (0.004) |  | (0.005) |
|  |  | [-0.020] |  | [-0.341] |  | [-0.037] |  | [-0.167] |  | [-0.187] |
| $\chi^{2}$ test | . | 0.6 | . | 240.6*** | . | 4.4** | . | 251.2*** | . | 297.6*** |
| Adj. R^2^ | 0.062 | 0.062 | 0.077 | 0.095 | 0.090 | 0.091 | 0.080 | 0.096 | 0.093 | 0.118 |
| Obs. | 15,234 | 15,234 | 15,178 | 15,178 | 2,319 | 2,319 | 15,164 | 15,164 | 12,154 | 12,154 |
| **Panel C: Mental Health and Wellbeing** | | | | | | | | | | |
|  | MCS | | Mental health | | Psychological distress | | Life satisfaction | | Health satisfaction | |
| LOC | 0.603*** | 0.542*** | 10.839*** | 9.791*** | -0.199*** | -0.181*** | 0.647*** | 0.593*** | 0.774*** | 0.673*** |
|  | (0.007) | (0.008) | (0.133) | (0.146) | (0.003) | (0.003) | (0.013) | (0.014) | (0.017) | (0.018) |
|  | . | . | [0.150] | [0.135] | [-0.997] | [-0.908] | [0.081] | [0.074] | [0.108] | [0.094] |
| SC |  | 0.166*** |  | 2.836*** |  | -0.048*** |  | 0.148*** |  | 0.273*** |
|  |  | (0.008) |  | (0.138) |  | (0.003) |  | (0.012) |  | (0.017) |
|  |  | . |  | [0.039] |  | [-0.241] |  | [0.019] |  | [0.038] |
| $\chi^{2}$ test | . | 375.3*** | . | 351.9*** | . | 205.7*** | . | 140.2*** | . | 236.5*** |
| Adj. R^2^ | 0.395 | 0.416 | 0.388 | 0.407 | 0.283 | 0.294 | 0.224 | 0.232 | 0.199 | 0.215 |
| Obs. | 15,000 | 15,000 | 15,233 | 15,233 | 15,256 | 15,256 | 15,283 | 15,283 | 15,281 | 15,281 |
| *Notes:* HILDA wave 19, analysis sample. OLS regressions. Locus of control and self-control are constructed as factor scores derived from two separate factor analyses. All regressions control for gender, age (in categories), education (in categories), migrant status, Indigenous status, state of residence fixed effects and a constant. Robust standard errors in parentheses. Relative effect sizes, where relevant, are in brackets. $\chi^{2}$ test is a test of the null hypothesis that the locus of control coefficients from the initial (without self-control) and subsequent (with self-control) model are not statistically different from each other. * p<0.1, ** p<0.05, *** p<0.01. | | | | | | | | | | |

**Table A.13: Health outcomes regressed on locus of control and self-control, using SCQ weights**

|  | (1) | (2) | (3) | (4) | (5) | (6) | (7) | (8) | (9) | (10) |
| --- | --- | --- | --- | --- | --- | --- | --- | --- | --- | --- |
| **Panel A: Overall and Physical Health** | | | | | | | | | | |
|  | Self-rated health | | PCS | | General health | | Overweight | | Obese | |
| LOC | 0.356*** | 0.301*** | 0.266*** | 0.254*** | 9.904*** | 8.362*** | -0.005 | 0.015** | -0.027*** | -0.010* |
|  | (0.011) | (0.012) | (0.011) | (0.013) | (0.241) | (0.262) | (0.006) | (0.006) | (0.005) | (0.005) |
|  | [0.106] | [0.090] | . | . | [0.150] | [0.126] | [-0.009] | [0.024] | [-0.103] | [-0.040] |
| SC |  | 0.156*** |  | 0.035*** |  | 4.357*** |  | -0.056*** |  | -0.046*** |
|  |  | (0.011) |  | (0.012) |  | (0.239) |  | (0.006) |  | (0.005) |
|  |  | [0.046] |  | . |  | [0.066] |  | [-0.094] |  | [-0.179] |
| $\chi^{2}$ test | . | 166.7*** | . | 9.2*** | . | 249.7*** | . | 77.1*** | . | 72.7*** |
| Adj. R^2^ | 0.251 | 0.272 | 0.282 | 0.283 | 0.276 | 0.309 | 0.074 | 0.084 | 0.052 | 0.061 |
| Obs. | 14,991 | 14,991 | 14,806 | 14,806 | 14,953 | 14,953 | 14,622 | 14,622 | 14,622 | 14,622 |
| **Panel B: Health Behaviors** | | | | | | | | | | |
|  | Inactive | | Smoking | | Number of cigarettes | | Alcohol: weekly | | Alcohol: 3+ drinks | |
| LOC | -0.051*** | -0.052*** | -0.022*** | -0.005 | -3.689** | -2.586 | 0.053*** | 0.078*** | 0.008 | 0.039*** |
|  | (0.005) | (0.005) | (0.004) | (0.004) | (1.656) | (1.623) | (0.006) | (0.006) | (0.006) | (0.006) |
|  | [-0.405] | [-0.417] | [-0.147] | [-0.033] | [-0.052] | [-0.036] | [0.133] | [0.195] | [0.017] | [0.080] |
| SC |  | 0.004 |  | -0.047*** |  | -3.493** |  | -0.070*** |  | -0.089*** |
|  |  | (0.004) |  | (0.004) |  | (1.743) |  | (0.006) |  | (0.006) |
|  |  | [0.033] |  | [-0.322] |  | [-0.049] |  | [-0.175] |  | [-0.184] |
| $\chi^{2}$ test | . | 1.0 | . | 104.9*** | . | 3.8* | . | 135.7*** | . | 169.9*** |
| Adj. R^2^ | 0.063 | 0.063 | 0.067 | 0.081 | 0.101 | 0.104 | 0.093 | 0.109 | 0.107 | 0.132 |
| Obs. | 15,035 | 15,035 | 14,982 | 14,982 | 2,284 | 2,284 | 14,965 | 14,965 | 12,018 | 12,018 |
| **Panel C: Mental Health and Wellbeing** | | | | | | | | | | |
|  | MCS | | Mental health | | Psychological distress | | Life satisfaction | | Health satisfaction | |
| LOC | 0.571*** | 0.507*** | 10.348*** | 9.223*** | -0.200*** | -0.182*** | 0.631*** | 0.579*** | 0.735*** | 0.637*** |
|  | (0.011) | (0.012) | (0.201) | (0.223) | (0.005) | (0.005) | (0.018) | (0.020) | (0.022) | (0.024) |
|  | . | . | [0.144] | [0.128] | [-0.959] | [-0.871] | [0.079] | [0.073] | [0.102] | [0.088] |
| SC |  | 0.181*** |  | 3.175*** |  | -0.052*** |  | 0.146*** |  | 0.278*** |
|  |  | (0.011) |  | (0.196) |  | (0.005) |  | (0.017) |  | (0.021) |
|  |  | . |  | [0.044] |  | [-0.248] |  | [0.018] |  | [0.039] |
| $\chi^{2}$ test | . | 227.7*** | . | 221.3*** | . | 116.7*** | . | 73.9*** | . | 155.1*** |
| Adj. R^2^ | 0.361 | 0.388 | 0.359 | 0.384 | 0.274 | 0.287 | 0.220 | 0.228 | 0.200 | 0.217 |
| Obs. | 14,806 | 14,806 | 15,035 | 15,035 | 15,056 | 15,056 | 15,083 | 15,083 | 15,081 | 15,081 |
| *Notes:* HILDA wave 19, analysis sample. Weighted OLS regressions, using self-completion questionnaire weights. All regressions control for gender, age (in categories), education (in categories), migrant status, Indigenous status, state of residence fixed effects and a constant. Robust standard errors in parentheses. Relative effect sizes, where relevant, are in brackets. $\chi^{2}$ test is a test of the null hypothesis that the locus of control coefficients from the initial (without self-control) and subsequent (with self-control) model are not statistically different from each other. * p<0.1, ** p<0.05, *** p<0.01. | | | | | | | | | | |

**Table A.14: Health outcomes regressed on internal locus of control, self-control, and their interaction, controlling for Big Five personality traits**

|  | (1) | (2) | (3) | (4) | (5) |
| --- | --- | --- | --- | --- | --- |
| **Panel A: Overall and Physical Health** | | | | | |
|  | Self-rated health | PCS | General health | Overweight | Obese |
| SC | 0.097*** | -0.022 | 3.196*** | -0.040*** | -0.039*** |
|  | (0.018) | (0.020) | (0.408) | (0.009) | (0.009) |
|  | [0.029] | . | [0.048] | [-0.066] | [-0.145] |
| Internal LOC | 0.514*** | 0.519*** | 14.403*** | -0.009 | -0.041*** |
|  | (0.020) | (0.023) | (0.460) | (0.011) | (0.010) |
|  | [0.154] | . | [0.218] | [-0.015] | [-0.154] |
| SC*Internal LOC | 0.096*** | 0.121*** | 2.061*** | -0.024** | -0.021** |
|  | (0.020) | (0.022) | (0.437) | (0.010) | (0.010) |
|  | [0.029] | . | [0.031] | [-0.040] | [-0.078] |
| Adj. R^2^ | 0.238 | 0.268 | 0.265 | 0.075 | 0.061 |
| Obs. | 13,279 | 13,120 | 13,240 | 12,983 | 12,983 |
| **Panel B: Health Behaviors** | | | | | |
|  | Inactive | Smoking | Number of cigarettes | Alcohol: weekly | Alcohol: 3+ drinks |
| SC | -0.004 | -0.056*** | -2.389 | -0.071*** | -0.085*** |
|  | (0.008) | (0.007) | (2.442) | (0.009) | (0.010) |
|  | [-0.035] | [-0.353] | [-0.034] | [-0.164] | [-0.176] |
| Internal LOC | -0.101*** | -0.027*** | -6.113* | 0.146*** | 0.069*** |
|  | (0.008) | (0.008) | (3.413) | (0.010) | (0.012) |
|  | [-0.838] | [-0.169] | [-0.086] | [0.338] | [0.143] |
| SC*Internal LOC | -0.008 | 0.004 | -0.282 | 0.006 | -0.002 |
|  | (0.008) | (0.008) | (2.748) | (0.010) | (0.011) |
|  | [-0.069] | [0.024] | [-0.004] | [0.014] | [-0.004] |
| Adj. R^2^ | 0.057 | 0.098 | 0.092 | 0.083 | 0.127 |
| Obs. | 13,315 | 13,269 | 1,902 | 13,252 | 10,867 |
| **Panel C: Mental Health and Wellbeing** | | | | | |
|  | MCS | Mental health | Psychological distress | Life satisfaction | Health satisfaction |
| SC | 0.222*** | 3.858*** | -0.100*** | 0.166*** | 0.171*** |
|  | (0.020) | (0.365) | (0.008) | (0.033) | (0.043) |
|  | . | [0.053] | [-0.500] | [0.021] | [0.024] |
| Internal LOC | 0.872*** | 15.484*** | -0.303*** | 0.894*** | 1.188*** |
|  | (0.022) | (0.390) | (0.010) | (0.034) | (0.047) |
|  | . | [0.214] | [-1.518] | [0.112] | [0.166] |
| SC*Internal LOC | -0.033 | -0.794** | 0.068*** | 0.015 | 0.167*** |
|  | (0.021) | (0.378) | (0.008) | (0.034) | (0.045) |
|  | . | [-0.011] | [0.341] | [0.002] | [0.023] |
| Adj. R^2^ | 0.359 | 0.354 | 0.273 | 0.196 | 0.194 |
| Obs. | 13,120 | 13,314 | 13,333 | 13,357 | 13,354 |
| *Notes:* HILDA wave 19, analysis sample. OLS regressions. In addition, all regressions control for gender, age (in categories), education (in categories), migrant status, Indigenous status, standardized measures of each Big Five personality trait (openness to new experiences, conscientiousness, extraversion, agreeableness, emotional stability), as well as a maximum set of fixed effects for the state of residence and a constant. Robust standard errors in parentheses. Relative effect sizes, where relevant, are in brackets. * p<0.1, ** p<0.05, *** p<0.01. | | | | | |

**Table A.15: Health outcomes regressed on average internal locus of control, self-control, and their interaction**

|  | (1) | (2) | (3) | (4) | (5) |
| --- | --- | --- | --- | --- | --- |
| **Panel A: Overall and Physical Health** | | | | | |
|  | Self-rated health | PCS | General health | Overweight | Obese |
| SC | 0.143*** | 0.014 | 4.460*** | -0.048*** | -0.047*** |
|  | (0.017) | (0.019) | (0.382) | (0.008) | (0.008) |
|  | [0.043] | . | [0.067] | [-0.078] | [-0.179] |
| Internal LOC | 0.486*** | 0.467*** | 14.068** | -0.009 | -0.034*** |
|  | (0.019) | (0.022) | (0.436) | (0.010) | (0.009) |
|  | [0.145] | . | [0.212] | [-0.015] | [0.127] |
| SC*Internal LOC | 0.094*** | 0.091*** | 1.858*** | -0.020** | -0.010 |
|  | (0.018) | (0.020) | (0.418) | (0.010) | (0.009) |
|  | [0.028] | . | [0.028] | [-0.032] | [-0.039] |
| Adj. R^2^ | 0.218 | 0.258 | 0.237 | 0.082 | 0.057 |
| Obs. | 15,189 | 15,000 | 15,151 | 14,812 | 14,812 |
| **Panel B: Health Behaviors** | | | | | |
|  | Inactive | Smoking | Number of cigarettes | Alcohol: weekly | Alcohol: 3+ drinks |
| SC | -0.004 | -0.064*** | -1.998 | -0.075*** | -0.090*** |
|  | (0.007) | (0.007) | (2.202) | (0.008) | (0.009) |
|  | [-0.034] | [-0.402] | [-0.028] | [-0.175] | [-0.186] |
| Internal LOC | -0.082*** | -0.031*** | -4.399 | 0.148*** | 0.061*** |
|  | (0.008) | (0.007) | (3.188) | (0.009) | (0.011) |
|  | [-0.686] | [-0.195] | [-0.062] | [0.343] | [0.126] |
| SC*Internal LOC | -0.012 | 0.013* | -1.082 | 0.021** | 0.011 |
|  | (0.008) | (0.008) | (2.568) | (0.009) | (0.010) |
|  | [-0.101] | [0.082] | [-0.015] | [0.048] | [0.023] |
| Adj. R^2^ | 0.053 | 0.097 | 0.099 | 0.092 | 0.116 |
| Obs. | 15,234 | 15,178 | 2,319 | 15,164 | 12,154 |
| **Panel C: Mental Health and Wellbeing** | | | | | |
|  | MCS | Mental health | Psychological distress | Life satisfaction | Health satisfaction |
| SC | 0.280*** | 4.844*** | -0.114*** | 0.215*** | 0.265*** |
|  | (0.019) | (0.334) | (0.008) | (0.030) | (0.039) |
|  | . | [0.067] | [-0.570] | [0.027] | [0.037] |
| Internal LOC | 0.858*** | 15.603*** | -0.309*** | 0.966*** | 1.142*** |
|  | (0.021) | (0.375) | (0.009) | (0.033) | (0.044) |
|  | . | [0.216] | [-1.549] | [0.121] | [0.159] |
| SC*Internal LOC | -0.016 | -0.244 | 0.050*** | 0.059* | 0.181*** |
|  | (0.020) | (0.361) | (0.008) | (0.032) | (0.042) |
|  | . | [-0.003] | [0.248] | [0.007] | [0.025] |
| Adj. R^2^ | 0.296 | 0.289 | 0.234 | 0.162 | 0.169 |
| Obs. | 15,000 | 15,233 | 15,256 | 15,283 | 15,281 |
| *Notes:* HILDA wave 19, analysis sample. OLS regressions. LOC is constructed here as the average locus of control score for all relevant waves an individual is observed in the HILDA longitudinal file. In addition, all regressions control for gender, age (in categories), education (in categories), migrant status, Indigenous status, as well as a maximum set of fixed effects for the state of residence and a constant. Robust standard errors in parentheses. Relative effect sizes, where relevant, are in brackets. * p<0.1, ** p<0.05, *** p<0.01. | | | | | |

**Table A.16: Health outcomes regressed on internal locus of control (instrumented), self-control, and their interaction**

|  | (1) | (2) | (3) | (4) | (5) |
| --- | --- | --- | --- | --- | --- |
| **Panel A: Overall and Physical Health** | | | | | |
|  | Self-rated health | PCS | General health | Overweight | Obese |
| SC | -0.037 | -0.106** | -0.616 | -0.032 | -0.040** |
|  | (0.043) | (0.048) | (1.003) | (0.021) | (0.020) |
|  | [-0.011] | . | [-0.009] | [-0.052] | [-0.150] |
| Internal LOC | 1.149*** | 1.075*** | 33.178*** | -0.011 | -0.112*** |
|  | (0.059) | (0.065) | (1.379) | (0.030) | (0.029) |
|  | [0.344] | . | [0.501] | [-0.017] | [-0.416] |
| SC*Internal LOC | 0.225*** | 0.162*** | 5.401*** | -0.043* | -0.012 |
|  | (0.052) | (0.057) | (1.206) | (0.026) | (0.025) |
|  | [0.067] | . | [0.082] | [-0.071] | [-0.044] |
| Adj. R^2^ | 0.173 | 0.217 | 0.148 | 0.062 | 0.049 |
| Obs. | 11,931 | 11,789 | 11,897 | 11,689 | 11,689 |
| **Panel B: Health Behaviors** | | | | | |
|  | Inactive | Smoking | Number of cigarettes | Alcohol: weekly | Alcohol: 3+ drinks |
| SC | 0.005 | -0.042*** | 5.578 | -0.146*** | -0.113*** |
|  | (0.018) | (0.016) | (6.720) | (0.021) | (0.023) |
|  | [0.046] | [-0.264] | [0.078] | [-0.340] | [-0.233] |
| Internal LOC | -0.190*** | -0.114*** | -24.275** | 0.418*** | 0.165*** |
|  | (0.023) | (0.023) | (11.526) | (0.031) | (0.035) |
|  | [-1.580] | [-0.713] | [-0.341] | [0.971] | [0.342] |
| SC*Internal LOC | -0.005 | 0.002 | -9.068 | 0.072*** | 0.022 |
|  | (0.021) | (0.020) | (9.292) | (0.026) | (0.028) |
|  | [-0.039] | [0.014] | [-0.127] | [0.166] | [0.045] |
| Adj. R^2^ | 0.040 | 0.080 | 0.064 | 0.027 | 0.108 |
| Obs. | 11,967 | 11,926 | 1,701 | 11,905 | 9,789 |
| **Panel C: Mental Health and Wellbeing** | | | | | |
|  | MCS | Mental health | Psychological distress | Life satisfaction | Health satisfaction |
| SC | 0.063 | 0.766 | -0.069*** | -0.125* | -0.107 |
|  | (0.046) | (0.842) | (0.018) | (0.070) | (0.099) |
|  | . | [0.011] | [-0.347] | [-0.016] | [-0.015] |
| Internal LOC | 1.798*** | 33.192*** | -0.634*** | 2.154*** | 2.686*** |
|  | (0.063) | (1.154) | (0.026) | (0.096) | (0.132) |
|  | . | [0.459] | [-3.173] | [0.270] | [0.374] |
| SC*Internal LOC | 0.098* | 1.967* | 0.060*** | 0.293*** | 0.407*** |
|  | (0.055) | (1.011) | (0.022) | (0.083) | (0.118) |
|  | . | [0.027] | [0.300] | [0.037] | [0.057] |
| Adj. R^2^ | 0.220 | 0.197 | 0.151 | 0.076 | 0.100 |
| Obs. | 11,789 | 11,967 | 11,976 | 11,998 | 11,998 |
| *Notes:* HILDA wave 19, analysis sample. 2SLS regressions. Internal LOC is an individual’s 2019 locus of control score in the top quartile, which is instrumented by the individual’s 2015 locus of control score being in the top quartile. In addition, all regressions control for gender, age (in categories), education (in categories), migrant status, Indigenous status, as well as a maximum set of fixed effects for the state of residence and a constant. Robust standard errors in parentheses. Relative effect sizes, where relevant, are in brackets. * p<0.1, ** p<0.05, *** p<0.01. | | | | | |

**Table A.17: Health outcomes regressed on internal locus of control, self-control, and their interaction, using factor scores for locus of control and self-control**

|  | (1) | (2) | (3) | (4) | (5) |
| --- | --- | --- | --- | --- | --- |
| **Panel A: Overall and Physical Health** | | | | | |
|  | Self-rated health | PCS | General health | Overweight | Obese |
| SC | 0.125*** | -0.012 | 3.831*** | -0.036*** | -0.037*** |
|  | (0.016) | (0.018) | (0.369) | (0.008) | (0.008) |
|  | [0.037] | . | [0.058] | [-0.059] | [-0.139] |
| Internal LOC | 0.556*** | 0.498*** | 15.636*** | -0.010 | -0.043*** |
|  | (0.019) | (0.022) | (0.435) | (0.010) | (0.009) |
|  | [0.166] | . | [0.236] | [-0.016] | [-0.159] |
| SC*Internal LOC | 0.101*** | 0.120*** | 2.314*** | -0.035*** | -0.021** |
|  | (0.018) | (0.020) | (0.408) | (0.009) | (0.009) |
|  | [0.030] | . | [0.035] | [-0.057] | [-0.080] |
| Adj. R^2^ | 0.228 | 0.261 | 0.250 | 0.081 | 0.055 |
| Obs. | 15,189 | 15,000 | 15,151 | 14,812 | 14,812 |
| **Panel B: Health Behaviors** | | | | | |
|  | Inactive | Smoking | Number of cigarettes | Alcohol: weekly | Alcohol: 3+ drinks |
| SC | 0.001 | -0.057*** | -3.243 | -0.068*** | -0.084*** |
|  | (0.007) | (0.007) | (2.035) | (0.008) | (0.009) |
|  | [0.007] | [-0.356] | [-0.046] | [-0.157] | [-0.174] |
| Internal LOC | -0.106*** | -0.033*** | -2.398 | 0.144*** | 0.068*** |
|  | (0.008) | (0.007) | (3.003) | (0.009) | (0.011) |
|  | [-0.882] | [-0.205] | [-0.034] | [0.335] | [0.141] |
| SC*Internal LOC | -0.013* | 0.003 | 0.565 | 0.009 | 0.000 |
|  | (0.008) | (0.008) | (2.434) | (0.009) | (0.010) |
|  | [-0.107] | [0.022] | [0.008] | [0.020] | [0.001] |
| Adj. R^2^ | 0.058 | 0.095 | 0.091 | 0.091 | 0.116 |
| Obs. | 15,234 | 15,178 | 2,319 | 15,164 | 12,154 |
| **Panel C: Mental Health and Wellbeing** | | | | | |
|  | MCS | Mental health | Psychological distress | Life satisfaction | Health satisfaction |
| SC | 0.257*** | 4.561*** | -0.114*** | 0.203*** | 0.229*** |
|  | (0.018) | (0.326) | (0.007) | (0.030) | (0.038) |
|  | . | [0.063] | [-0.571] | [0.025] | [0.032] |
| Internal LOC | 1.014*** | 18.177*** | -0.354*** | 1.066*** | 1.281*** |
|  | (0.021) | (0.373) | (0.009) | (0.033) | (0.044) |
|  | . | [0.251] | [-1.774] | [0.134] | [0.179] |
| SC*Internal LOC | -0.016 | -0.375 | 0.058*** | 0.054* | 0.200*** |
|  | (0.020) | (0.352) | (0.008) | (0.032) | (0.041) |
|  | . | [-0.005] | [0.292] | [0.007] | [0.028] |
| Adj. R^2^ | 0.345 | 0.335 | 0.273 | 0.178 | 0.182 |
| Obs. | 15,000 | 15,233 | 15,256 | 15,283 | 15,281 |
| *Notes:* HILDA wave 19, analysis sample. OLS regressions. Locus of control and self-control are constructed as factor scores derived from two separate factor analyses. In addition, all regressions control for gender, age (in categories), education (in categories), migrant status, Indigenous status, as well as a maximum set of fixed effects for the state of residence and a constant. Robust standard errors in parentheses. Relative effect sizes, where relevant, are in brackets. * p<0.1, ** p<0.05, *** p<0.01. | | | | | |

**Table A.18: Health outcomes regressed on internal locus of control, self-control, and their interaction, using SCQ weights**

|  | (1) | (2) | (3) | (4) | (5) |
| --- | --- | --- | --- | --- | --- |
| **Panel A: Overall and Physical Health** | | | | | |
|  | Self-rated health | PCS | General health | Overweight | Obese |
| SC | 0.118*** | -0.047* | 3.649*** | -0.027** | -0.038*** |
|  | (0.022) | (0.029) | (0.530) | (0.011) | (0.010) |
|  | [0.035] | . | [0.055] | [-0.046] | [-0.146] |
| Internal LOC | 0.567*** | 0.526*** | 15.727*** | 0.020 | -0.021* |
|  | (0.027) | (0.032) | (0.602) | (0.015) | (0.012) |
|  | [0.169] | . | [0.238] | [0.033] | [-0.080] |
| SC*Internal LOC | 0.110*** | 0.155*** | 2.596*** | -0.036*** | -0.014 |
|  | (0.024) | (0.030) | (0.578) | (0.013) | (0.011) |
|  | [0.033] | . | [0.039] | [-0.060] | [-0.054] |
| Adj. R^2^ | 0.246 | 0.273 | 0.266 | 0.085 | 0.061 |
| Obs. | 14,991 | 14,806 | 14,953 | 14,622 | 14,622 |
| **Panel B: Health Behaviors** | | | | | |
|  | Inactive | Smoking | Number of cigarettes | Alcohol: weekly | Alcohol: 3+ drinks |
| SC | 0.012 | -0.049*** | -5.459* | -0.065*** | -0.081*** |
|  | (0.010) | (0.008) | (2.859) | (0.009) | (0.011) |
|  | [0.099] | [-0.330] | [-0.077] | [-0.163] | [-0.168] |
| Internal LOC | -0.103*** | -0.018* | -3.972 | 0.154*** | 0.068*** |
|  | (0.012) | (0.009) | (3.974) | (0.012) | (0.015) |
|  | [-0.828] | [-0.123] | [-0.056] | [0.387] | [0.142] |
| SC*Internal LOC | -0.020* | 0.002 | 2.444 | 0.007 | -0.003 |
|  | (0.011) | (0.009) | (3.389) | (0.011) | (0.013) |
|  | [-0.164] | [0.016] | [0.034] | [0.017] | [-0.006] |
| Adj. R^2^ | 0.058 | 0.082 | 0.104 | 0.104 | 0.130 |
| Obs. | 15,035 | 14,982 | 2,284 | 14,965 | 12,018 |
| **Panel C: Mental Health and Wellbeing** | | | | | |
|  | MCS | Mental health | Psychological distress | Life satisfaction | Health satisfaction |
| SC | 0.244*** | 4.436*** | -0.109*** | 0.217*** | 0.201*** |
|  | (0.023) | (0.408) | (0.011) | (0.041) | (0.047) |
|  | . | [0.062] | [-0.521] | [0.027] | [0.028] |
| Internal LOC | 0.950*** | 17.254*** | -0.364*** | 1.013*** | 1.198*** |
|  | (0.028) | (0.496) | (0.013) | (0.045) | (0.055) |
|  | . | [0.239] | [-1.747] | [0.128] | [0.166] |
| SC*Internal LOC | 0.009 | 0.011 | 0.049*** | 0.026 | 0.231*** |
|  | (0.025) | (0.444) | (0.012) | (0.043) | (0.051) |
|  | . | [0.000] | [0.237] | [0.003] | [0.032] |
| Adj. R^2^ | 0.323 | 0.321 | 0.271 | 0.174 | 0.186 |
| Obs. | 14,806 | 15,035 | 15,056 | 15,083 | 15,081 |
| *Notes:* HILDA wave 19, analysis sample. Weighted OLS regressions, using self-completion questionnaire weights. In addition, all regressions control for gender, age (in categories), education (in categories), migrant status, Indigenous status, as well as a maximum set of fixed effects for the state of residence and a constant. Robust standard errors in parentheses. Relative effect sizes, where relevant, are in brackets. * p<0.1, ** p<0.05, *** p<0.01. | | | | | |

**Table A.19: Health outcomes regressed on internal locus of control (defined via 10^th^ percentile threshold), self-control, and their interaction**

|  | (1) | (2) | (3) | (4) | (5) |
| --- | --- | --- | --- | --- | --- |
| **Panel A: Overall and Physical Health** | | | | | |
|  | Self-rated health | PCS | General health | Overweight | Obese |
| SC | 0.129*** | -0.026 | 3.598*** | -0.024** | -0.032*** |
|  | (0.024) | (0.028) | (0.556) | (0.012) | (0.011) |
|  | [0.039] | . | [0.054] | [-0.040] | [-0.118] |
| Internal LOC | 0.644*** | 0.582*** | 17.758*** | -0.003 | -0.039*** |
|  | (0.029) | (0.034) | (0.677) | (0.014) | (0.013) |
|  | [0.193] | . | [0.268] | [-0.005] | [-0.145] |
| SC*Internal LOC | 0.114*** | 0.146*** | 3.189*** | -0.045*** | -0.030*** |
|  | (0.025) | (0.029) | (0.578) | (0.012) | (0.012) |
|  | [0.034] | . | [0.048] | [-0.075] | [-0.112] |
| Adj. R^2^ | 0.214 | 0.250 | 0.224 | 0.082 | 0.056 |
| Obs. | 15,189 | 15,000 | 15,151 | 14,812 | 14,812 |
| **Panel B: Health Behaviors** | | | | | |
|  | Inactive | Smoking | Number of cigarettes | Alcohol: weekly | Alcohol: 3+ drinks |
| SC | 0.002 | -0.060*** | -2.800 | -0.073*** | -0.086*** |
|  | (0.010) | (0.009) | (2.890) | (0.011) | (0.012) |
|  | [0.015] | [-0.373] | [-0.039] | [-0.170] | [-0.177] |
| Internal LOC | -0.123*** | -0.026** | -1.477 | 0.162*** | 0.088*** |
|  | (0.012) | (0.010) | (4.218) | (0.013) | (0.016) |
|  | [-1.022] | [-0.161] | [-0.021] | [0.377] | [0.183] |
| SC*Internal LOC | -0.019* | 0.003 | -0.350 | 0.023** | 0.006 |
|  | (0.011) | (0.010) | (3.144) | (0.011) | (0.013) |
|  | [-0.160] | [0.020] | [-0.005] | [0.054] | [0.013] |
| Adj. R^2^ | 0.054 | 0.095 | 0.090 | 0.086 | 0.115 |
| Obs. | 15,234 | 15,178 | 2,319 | 15,164 | 12,154 |
| **Panel C: Mental Health and Wellbeing** | | | | | |
|  | MCS | Mental health | Psychological distress | Life satisfaction | Health satisfaction |
| SC | 0.263*** | 4.765*** | -0.108*** | 0.181*** | 0.220*** |
|  | (0.025) | (0.458) | (0.010) | (0.046) | (0.057) |
|  | . | [0.066] | [-0.541] | [0.023] | [0.031] |
| Internal LOC | 1.156*** | 20.528*** | -0.413*** | 1.336*** | 1.526*** |
|  | (0.031) | (0.567) | (0.014) | (0.055) | (0.069) |
|  | . | [0.284] | [-2.068] | [0.168] | [0.213] |
| SC*Internal LOC | 0.035 | 0.450 | 0.025** | 0.120** | 0.248*** |
|  | (0.026) | (0.475) | (0.010) | (0.047) | (0.058) |
|  | . | [0.006] | [0.125] | [0.015] | [0.035] |
| Adj. R^2^ | 0.292 | 0.281 | 0.227 | 0.163 | 0.165 |
| Obs. | 15,000 | 15,233 | 15,256 | 15,283 | 15,281 |
| *Notes:* HILDA wave 19, analysis sample. OLS regressions. In addition, all regressions control for gender, age (in categories), education (in categories), migrant status, Indigenous status, as well as a maximum set of fixed effects for the state of residence and a constant. Robust standard errors in parentheses. Relative effect sizes, where relevant, are in brackets. * p<0.1, ** p<0.05, *** p<0.01. | | | | | |

**Table A.20: Health outcomes regressed on internal locus of control (defined via median threshold), self-control, and their interaction**

|  | (1) | (2) | (3) | (4) | (5) |
| --- | --- | --- | --- | --- | --- |
| **Panel A: Overall and Physical Health** | | | | | |
|  | Self-rated health | PCS | General health | Overweight | Obese |
| SC | 0.192*** | 0.065*** | 5.525*** | -0.052*** | -0.052*** |
|  | (0.012) | (0.012) | (0.258) | (0.006) | (0.006) |
|  | [0.057] | . | [0.083] | [-0.086] | [-0.193] |
| Internal LOC | 0.419*** | 0.361*** | 12.463*** | 0.003 | -0.035*** |
|  | (0.015) | (0.015) | (0.321) | (0.008) | (0.008) |
|  | [0.125] | . | [0.188] | [0.004] | [-0.129] |
| SC*Internal LOC | 0.028* | 0.034** | 0.182 | -0.022*** | -0.005 |
|  | (0.015) | (0.015) | (0.330) | (0.008) | (0.007) |
|  | [0.008] | . | [0.003] | [-0.036] | [-0.020] |
| Adj. R^2^ | 0.220 | 0.253 | 0.245 | 0.081 | 0.056 |
| Obs. | 15,189 | 15,000 | 15,151 | 14,812 | 14,812 |
| **Panel B: Health Behaviors** | | | | | |
|  | Inactive | Smoking | Number of cigarettes | Alcohol: weekly | Alcohol: 3+ drinks |
| SC | -0.014*** | -0.066*** | -2.860* | -0.055*** | -0.082*** |
|  | (0.005) | (0.005) | (1.603) | (0.006) | (0.006) |
|  | [-0.118] | [-0.411] | [-0.040] | [-0.128] | [-0.170] |
| Internal LOC | -0.061*** | -0.011* | -1.846 | 0.116*** | 0.072*** |
|  | (0.005) | (0.006) | (2.655) | (0.008) | (0.009) |
|  | [-0.510] | [-0.067] | [-0.026] | [0.269] | [0.149] |
| SC*Internal LOC | 0.001 | 0.017*** | -0.194 | -0.010 | -0.007 |
|  | (0.006) | (0.006) | (2.343) | (0.008) | (0.009) |
|  | [0.008] | [0.107] | [-0.003] | [-0.023] | [-0.014] |
| Adj. R^2^ | 0.050 | 0.095 | 0.090 | 0.089 | 0.117 |
| Obs. | 15,234 | 15,178 | 2,319 | 15,164 | 12,154 |
| **Panel C: Mental Health and Wellbeing** | | | | | |
|  | MCS | Mental health | Psychological distress | Life satisfaction | Health satisfaction |
| SC | 0.325*** | 5.639*** | -0.131*** | 0.298*** | 0.406*** |
|  | (0.013) | (0.228) | (0.005) | (0.019) | (0.025) |
|  | . | [0.078] | [-0.656] | [0.037] | [0.057] |
| Internal LOC | 0.758*** | 13.738*** | -0.244*** | 0.794*** | 0.922*** |
|  | (0.015) | (0.263) | (0.006) | (0.022) | (0.030) |
|  | . | [0.190] | [-1.222] | [0.010] | [0.129] |
| SC*Internal LOC | -0.135*** | -2.322*** | 0.104*** | -0.087*** | -0.022 |
|  | (0.015) | (0.274) | (0.006) | (0.023) | (0.032) |
|  | . | [-0.032] | [0.523] | [-0.011] | [-0.003] |
| Adj. R^2^ | 0.303 | 0.294 | 0.218 | 0.157 | 0.165 |
| Obs. | 15,000 | 15,233 | 15,256 | 15,283 | 15,281 |
| *Notes:* HILDA wave 19, analysis sample. OLS regressions. In addition, all regressions control for gender, age (in categories), education (in categories), migrant status, Indigenous status, as well as a maximum set of fixed effects for the state of residence and a constant. Robust standard errors in parentheses. Relative effect sizes, where relevant, are in brackets. * p<0.1, ** p<0.05, *** p<0.01. | | | | | |

**Figure A.1: Rotated factor loadings**


*Notes:* HILDA wave 19, analysis sample with 15,288 observations. Loadings from factor analysis restricted to two factors after oblique rotation.
